# Patterns of Technical Variation in Chimpanzee Termite Fishing Behavior in Mbam and Djerem National Park, Cameroon

**DOI:** 10.1002/ajp.70014

**Published:** 2025-03-02

**Authors:** Tyler C. Andres‐Bray, Ian Nichols, Tabitha Wilke, Macy Hafner, Abigail Jordan, Andrea Eysseric, Vivianna Borzym, Ekwoge E. Abwe, Bethan Morgan, Mary Katherine Gonder

**Affiliations:** ^1^ Center for the Advancement of STEM Teaching and Learning Excellence Drexel University Philadelphia Pennsylvania USA; ^2^ Cameroon Biodiversity Protection Program Yaoundé Cameroon; ^3^ Department of Forest Resources and Environmental Conservation Virginia Tech Blacksburg Virginia USA; ^4^ Department of Ecology and Conservation Biology Texas A&M University College Station Texas USA; ^5^ Department of Biological Sciences Drexel University Philadelphia Pennsylvania USA; ^6^ Cameroon Biodiversity Association Douala Cameroon; ^7^ San Diego Zoo Wildlife Alliance San Diego California USA

**Keywords:** Nigeria‐Cameroon chimpanzee, primate behavior, quantitative ethnography, termite fishing

## Abstract

Chimpanzees exhibit considerable inter‐ and intra‐community variation in cognitively complex tool use behaviors, often attributed to social, genetic, and environmental factors. Termite fishing is a well‐documented chimpanzee tool‐using behavior that has been the subject of comparative research exploring behavioral variation between chimpanzee communities. However, termite fishing in the Nigeria‐Cameroon chimpanzee (*Pan troglodytes ellioti*) has been historically underrepresented due to a lack of habituated populations. In this study, we used remote‐activated camera traps at several termite mounds for 3 years to study termite fishing near Ganga Research Station in central Cameroon. We aimed to (1) identify elemental variation in chimpanzee termite fishing techniques at Ganga, an understudied community of *P. t. ellioti*, and (2) compare termite fishing behaviors in the Ganga community among more well‐studied chimpanzee communities. We found 46 different combinations of behavioral elements representing termite fishing techniques used by Ganga chimpanzees (*n* = 9) across five termite mounds. The average technique was between three and four elements long (*x̄* = 3.673), and many chimpanzees had unique personal repertoires. Chimpanzees at Ganga shared the most behavioral similarities with two communities of savanna chimpanzees, Dindefelo and Kayan, and the nearby rainforest community of La Belgique in southern Cameroon. This behavioral similarity between Ganga chimpanzees, who inhabit a complex forest/savanna matrix, and two distant savanna‐dwelling communities suggests similar environmental contexts contribute to termite fishing similarity. These results add to comparative studies of termite fishing behavior and demonstrate the utility of quantitative ethnographic methodology in exploring chimpanzee behavioral variation.

## Summary

- We used motion‐activated camera traps to examine patterns of termite fishing behavior in the Ganga chimpanzee community of the understudied Nigeria‐Cameroon chimpanzee (*Pan troglodytes ellio/*) in central Cameroon.
- Ganga chimpanzees demonstrated a total of 46 unique combinations of behavioral elements representing termite fishing techniques, with similarity between individual repertoires following known patterns of social transmission in chimpanzee tool use.
- Leveraging quantitative ethnographic methods from the learning sciences, we identified cultural similarities in termite fishing between the Ganga community and other chimpanzee communities that may be related to habitat similarity.

## Graphical abstract

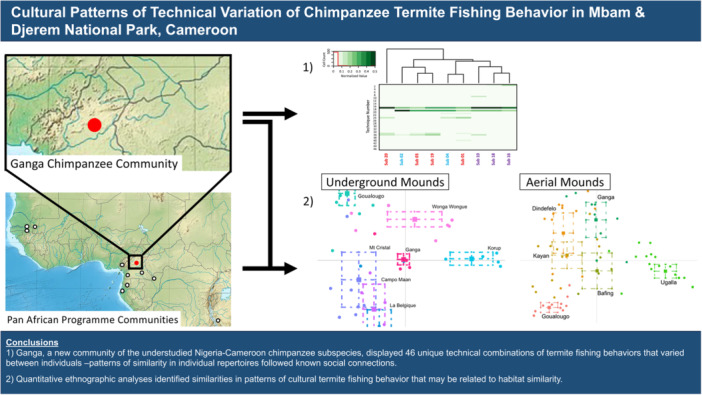

## Résumé

Les chimpanzés démontrent des niveaux élevés de variations intercommunautaires et intracommunautaires dans l'utilisation des outils cognitivement complexes, souvent attribués à des facteurs sociaux, génétiques et environnementaux. La pêche aux termites est un comportement bien documenté d'utilisation d'outils des chimpanzés qui a récemment fait l'objet de recherches comparatives explorant la variation comportmentale entre les communautés de chimpanzés. Cependant, la pêche aux termites chez le Nigeria‐Cameroon chimpanzé (*Pan troglodytes ellioti*) a été historiquement sous‐représentée à cause du manque de populations habituées. Dans cette projet, nous avons utilisé des pièges photographiques à plusieurs termitières pendant trois ans pour étudier la pêche aux termites à la station de recherche du Ganga, dans le centre du Cameroun. Notre objectif était: (1) identifier la variation élémentaire des techniques de pêche aux termites des chimpanzés à Ganga, une nouvelle communauté de *P. t. ellioti*, et (2) comparer ces comportements de pêche aux termites parmi les communautés de chimpanzés plus bien étudiées. Nous avons trouvé 46 combinaisons differents d'éléments comportementaux représentant les techniques de pêche aux termites utilisées par les chimpanzés du Ganga (*n* = 9) à cinq termitières. La technique moyenne mesurait entre 3 et 4 éléments (*x̄* = 3.673) et de nombreux chimpanzés avaient des répertoires personnels uniques. Les chimpanzés de Ganga présentaient des comportements très similaires à ceux de deux communautés de chimpanzés de savane, Dindefelo et Kayan, et à la communauté de La Belgique, dans le sud du Cameroun. Cette similitude comportementale entre les chimpanzés du Ganga, qui habitent une matrice complexe forêt/savane, et deux communautés éloignées vivant dans la savane suggère que des contextes environnementaux similaires contribuent à la similitude de la pêche aux termites. Ces résultats s'ajoutent aux études comparatives du comportement de pêche aux termites et démontrent l'utilité de la méthodologie ethnographique quantitative pour explorer les variations comportementale des chimpanzés.

**Abbreviations**
1h: one‐handed
CBPP: Cameroon Biodiversity Protection Program
ENA: Epistemic Network Analysis
MDNP: Mbam & Djerem National Park

## 1 Introduction

Tool use is a highly flexible, cognitively complex behavior that often allows chimpanzees to extend their ecological niche (Boesch et al. 2020; Fox et al. 1999; Sanz and Morgan 2011; Whiten et al. 1999). Defined as the modification/manipulation of an object to attain a goal (Goodall 1964), these behaviors allow access to high‐value diet items (e.g., social insects and nuts) that may provide nontrivial amounts of calories and micronutrients for chimpanzees (Deblauwe and Janssens 2008). These behaviors are comprised of distinct behavioral components (elements) that are strung together into meaningful, task‐oriented combinations (techniques) that may vary based on individual and ecological contexts (Boesch et al. 2020; Sanz and Morgan 2011).

Chimpanzee tool use varies considerably between individuals and between communities. Variation in the targets of tool use, the types of tools used, and the behavioral elements incorporated into tool use can be linked to ecological factors (McGrew and Collins 1985; Koops et al. 2013: Sanz and Morgan 2013), neutral genetic variation (Langergraber et al. 2011), and social/cultural factors (Whiten 2017), pointing to a complex interaction between genes, environment, and social context that shapes tool use. Increasingly in‐depth studies of chimpanzee behavior across many long‐term field studies have explored the role of these factors in shaping chimpanzee tool use variation and have demonstrated putatively cultural variation in tool use (Whiten et al. 1999). In this context, cultural variation refers to behavioral differences between communities arising predominantly via intergenerational and interindividual social transmission as opposed to genetic or environmental differences between those communities (Boesch et al. 2020; Whiten et al. 1999; Whiten 2005).

Many studies attempting to identify cultural variation in chimpanzee behavior use an exclusionary method to deem certain behavioral variants “cultural” if they differ between communities irrespective of ecological explanation, as the social learning involved in tool use development can lead to behavioral homogeneity within groups (Kendal et al. 2015; Whiten et al. 1999). For example, two nearby chimpanzee communities in Uganda used two different types of tools (sticks vs. leaf sponges) to acquire experimentally provided honey, despite both communities having access to sticks and leaves (Gruber et al. 2009). The assumption here is often that if both communities exhibit different behaviors despite having access to the same materials and existing within the same population (i.e., may exchange migrants and thus share genetic similarities), these behavioral differences are likely maintained by social transmission and represent cultural variation between neighboring chimpanzee groups. Whereas these studies frequently focus on differences in the types of behaviors present in a community, there may be variation in the ways chimpanzees employ the same types of tools to accomplish the same goals. In this way, studying the elemental variation of skilled behaviors like tool use is another potentially valuable method for studying chimpanzee culture (Byrne 2007). Tool‐using behaviors are visible expressions of goal‐directed cognition, meaning that chimpanzees make choices regarding tools and how they use them to acquire a resource, which can lead to variation in tool‐using repertoires as chimpanzees respond to specific environmental challenges (Andres‐Bray and Gonder 2024) with behaviors built on innovated, socially‐transmitted experiences (Lamon et al. 2017; Whiten et al. 1999) and potentially grounded in genetic predispositions (Langergraber et al. 2011). When exploring variation in complex behaviors, quantifying the elemental “building blocks” provides a means of estimating “skill relevant repertoires,” which can then allow for the comparison of socially driven elements between communities to approximate putative cultural differences (Boesch et al. 2020; Byrne 2007; Sanz and Morgan 2011).

Termite fishing is a well‐documented tool‐use behavior that is widespread among chimpanzee communities across Africa (Boesch et al. 2020; Whiten et al. 1999). This task involves the modification and insertion of a flexible plant probe into termite nests, exploiting the defensive biting behavior of termite soldiers to pull them out of the mounds (Goodall 1964; Sanz and Morgan 2011). There is considerable variation in technical combinations of behavioral elements, which we define as individual actions that when used together in sequence accomplish a specific goal, used in termite fishing, both between chimpanzee communities (Boesch et al. 2020) and between individual chimpanzees within the same communities (Sanz and Morgan 2011). Certain elements incorporated into this tool‐use behavior may be a response to ecological or termite prey characteristics, but others may be the result of individual preferences based on past experiences or founded on genetic predispositions that could then be socially transmitted within a community and contribute to community cultures.

Studies of termite fishing have been conducted in groups of chimpanzees in both wild and captive contexts. Captive studies of tool use behaviors have the benefit of increased control over the tool‐using context and have demonstrated innovation and social transmission of tool use in chimpanzees (e.g., Hopper et al. 2007; Paquette 1992). Wild studies of tool use, in contrast, have less control over the elements associated with chimpanzee tool use and may rely more heavily on opportunistic observations, but they provide a more natural, ecologically valid opportunity to explore tool use (Boesch et al. 2020; Koops et al. 2013; Sanz and Morgan 2013). An increasing number of wild chimpanzee communities, including some long‐term chimpanzee studies (e.g., Goualougo: Sanz and Morgan 2011; Gombe: Lonsdorf 2005; Issa Valley: Stewart and Piel 2014) and recently in several understudied communities (Boesch et al. 2020), have explored chimpanzee termite fishing; however, nearly all of these studies have focused on three of the four recognized chimpanzee subspecies: the western chimpanzee (*Pan troglodytes verus*), the central chimpanzee (*P. t. troglodytes*), and the eastern chimpanzee (*P. t. schweinfurthii*). To date, direct examinations of termite fishing behavioral elements in the Nigeria‐Cameroon chimpanzee (*P. t. ellioti)* have soley been done in a single chimpanzee community (Korup) in western Cameroon (Boesch et al. 2020). This limited observational exploration of *P. t. ellioti* tool use is due in part to their small population sizes and range (Oates et al. 2016), as well as a lack of habituated study groups, which makes direct observations nearly impossible. In this study, we used motion‐activated camera traps to examine termite fishing behavior in the Ganga community of *P. t. ellioti*, living in Mbam & Djerem National Park (MDNP) in central Cameroon. Specifically, this project addressed the following questions:

1. Is there interindividual variation in the techniques used by Ganga chimpanzees to fish for termites?
2. How similar is the termite fishing repertoire of Ganga chimpanzees to other, more well‐studied chimpanzee communities?

Chimpanzees at Ganga are known to fish for termites variably throughout the year (Andres‐Bray et al. 2024). Furthermore, recent research has found that Ganga chimpanzees have a higher proportion of animal matter in their diets relative to chimpanzees in Ebo Forest in western Cameroon, which suggests that insect prey like termites may be an important dietary resource in this community (Abwe et al. 2020). Furthermore, previous work in this region has determined that there are three genetically distinct populations of chimpanzees in Cameroon, a western rainforest population of *P. t. ellioti*, a central ecotone population of *P. t. ellioti*, and a southern population of *P. t. troglodytes* also inhabiting dense rainforests (Mitchell et al. 2015). This research also identified regular gene flow between the two *P. t. ellioti* populations, as well as gene flow to the degree of one migrant per generation from the *P. t. troglodytes* population in the south into the *P. t. ellioti* population in central Cameroon (Mitchell et al. 2015). This suggests that over many generations, there could be social transmission of termite fishing behavioral elements between communities within these different populations. Given the increasing number of chimpanzee communities where the behavioral elements involved in termite fishing have been explored, comparing sequences of elements used in termite fishing at Ganga to other chimpanzee communities can help elucidate how social and environmental factors shape termite fishing variation. Studies have shown a negative correlation between cultural similarity and geographic distance in humans (Jordan and Shennan 2003), chimpanzees (Kamilar and Marshack 2012), and orangutans (Van Schaik et al. 2003), irrespective of environmental similarity, which supports the role of social factors in shaping behavioral similarity as it suggests a diffusion process of similar behaviors between nearby communities through dispersal (Van Schaik et al. 2003). Additionally, exploring behavioral similarity related to environmental similarity can identify the role that environmental factors play in shaping behavioral similarity, though neither of these methods independently can exclude the effects of other factors, including genes, in shaping behavioral variation (Kalan et al. 2020; Kamilar and Marshack 2012). In this study, we used Epistemic Network Analysis (ENA), a quantitative ethnographic technique that analyzes patterns in cognitive/behavioral data, to compare termite fishing behaviors in Ganga chimpanzees with those of 10 other chimpanzee communities observed by Boesch et al. (2020). By doing this, we will be able to make potentially interesting inferences about associations between behavioral similarities, geographic distance, and broad environmental similarity with the Ganga community in this study.

## 2 Methods

### 2.1 Ethical Note

This study adheres to all guidelines in the American Society of Primatologists’ Principles of the Ethical Treatment of Primates. The following methodological protocols were reviewed by the IACUC committee at Drexel University and determined to be exempt due to the exclusive use of indirect observations via camera traps. This study was conducted with permission from the Ministry of Forests and Fauna (Permit N° 004/SL/EFG/D/DA/SES/Ka) in Cameroon and the conservator of MDNP (Permit N° 00000002/L/RC/DRFOF/PNMD/SC).

### 2.2 Data Collection

#### 2.2.1 Camera Trap Survey – Ganga

We opportunistically collected videos of chimpanzees participating in termite fishing using Browning Spec Ops Advantage camera traps placed at eight known termite mounds along transects near Ganga Research Station in MDNP (Figure S1). Camera traps were secured on trees at a distance of between 5 and 10 m from each mound using a rigger belt at a height between 1 and 1.5 m above the ground to provide adequate distance to capture behaviors of interest (Hedwig et al. 2018). Each camera trap was set to record for 20 s (low‐light conditions using infrared flash) or for 1‐min videos (full‐light conditions) when they detected motion, and to continue filming if motion continued. Silica packets were placed in each camera trap cover to reduce the effects of humidity on camera function. The CBPP biomonitoring team collected and replaced SD cards, batteries, and silica packets monthly, and ensured that cameras were functional.

Videos were collected from an initial pilot project by I. Nichols in May 2019 (Mound I) and then collected continuously at seven mounds from February 2020 to April 2023 (Mound A‐I), with an eighth mound added when it was identified in January 2021 (Mound J) (Table S1). Each mound was classified by mound shape (underground: *n* = 3, aerial: *n* = 5), where underground refers to a subterranean termite nest that has no protruding above‐ground structure, whereas an aerial mound denotes a termite nest that has a termite‐built structure extending above the ground (Table S1). A total of 15,305 videos containing 42 different species were collected over the course of this study, including several sightings of rare species for this area such as the Central African potto (*Perodicticus edwardsi*), the African golden cat (*Caracal aurata*), and the crowned monkey (*Cercopithecus pogonias*). Altogether, 900 videos contained chimpanzee termite fishing, which represents 8 h 38 m 11 s of recording time. Of the 41 identified chimpanzees in this community, 17 were seen performing termite fishing‐related behavioral elements (adult: *n* = 11, Juvenile: *n* = 3, infant: *n* = 3) at 5 mounds (Figure S1). Visitors were predominantly adult females (*n* = 8) with dependent offspring (*n* = 3), whereas juveniles (male: *n* = 1, female: *n* = 2) would fish for termites frequently on their own.

The specific termite species at each mound is unconfirmed but is suspected to be *Sphaerotermes sphaerothorax* or possibly *Microtermes calvus* from previous efforts to collect and identify the termite species through morphological characteristics (Andres‐Bray et al. 2024). Both *S. sphaerothorax* and *M. calvus* are members of the mound‐building Macrotermitinae family (Darlington 2021). *S. sphaerothorax* is known to construct epigeal nests, and whereas *Microtermes spp*. are often subterranean, they are known to coexist with epigeal nest‐dwelling termites (Darlington 2021; Wood 1988). To our knowledge, this would be the first report of a chimpanzee community predating upon *S. sphaerothorax*, as the most common termite prey of chimpanzees are *Macrotermes spp*. (McGrew 2014). Little is known about *S. sphaerothorax* behavior, and thus, the characteristics of this termite prey may contribute to intercommunity behavioral variation.

#### 2.2.2 Video Coding

Low‐quality videos, meaning videos where chimpanzee behaviors were completely unidentifiable and videos lasting less than 5 s total, were excluded from the coding process. This left 382 high‐quality videos of chimpanzee termite fishing behavior, which represents 5 h 45 m 54 s of observation time total. We used QuickTime Media Player and Microsoft Excel to record the behavioral elements of each chimpanzee subject in each high‐quality video. We used a comprehensive ethogram of 72 chimpanzee behavioral elements constructed from several chimpanzee behavioral studies, with a code labeled “Other” to capture any behaviors that were not defined in the ethogram (Table S2) (Nishida et al. 1999; Boesch et al. 2020). Each behavioral element was considered mutually exclusive, meaning that the beginning of one behavioral element was also the end of the previous element. We recorded the start time to the nearest quarter second, body position, and hand used for every behavioral element in sequence. Videos were coded by T. Andres‐Bray and a dedicated team of undergraduate researchers from Drexel University and Texas A&M University. All undergraduate researchers went through a period of training via co‐coding 10 videos with T. Andres‐Bray, followed by a testing period where all coder's independently coded 20 additional videos (roughly 5% of total videos coded) to test interrater reliability. Cohen (1960) was calculated to determine that there was sufficient agreement between undergraduate coders and T. Andres‐Bray in terms of both the behaviors identified and the starting time of each behavior. Undergraduate researchers all achieved *k* ≤ 0.6, which is considered good agreement in behavioral studies, on individually coded practice videos before coding behavioral data on their own (Fleiss et al. 2013).

#### 2.2.3 Pan African Program Data

Data on termite fishing behavior from 10 other chimpanzee communities were obtained from Boesch et al. (2020) (https://www.nature.com/articles/s41562-020-0890-1#Sec15) to compare to the termite fishing behavior of Ganga chimpanzees. These data were collected using camera trap videos as part of the Pan African Program: The Cultured Chimpanzee project, which examined termite fishing variation across 10 behaviorally understudied chimpanzee communities based on a minimum of 1 year of fieldwork (Table 1). Boesch coded these videos for the presence of behavioral elements inferred to be socially transmitted rather than resulting from responses to environmental context like mound structure. The published data from Boesch et al. (2020) thus represented coded presence/absence data of termite fishing behavioral elements largely assumed to be socially driven across each chimpanzee in each observation sequence, making them consistent with our coded data from videos collected at Ganga. Each behavioral element in the Boesch et al. (2020) study corresponded to a behavioral element used to code videos from the Ganga community (Table S2), which allowed for comparisons with the Ganga chimpanzee community to situate the termite fishing behavioral repertoire of Ganga chimpanzees in the larger chimpanzee termite fishing literature.

**Table 1:** Demographic information of data from all chimpanzee communities.

Community	Subspecies	Location	Habitat type	Individuals used in analysis	Observation time
Dindefelo	*P. t. verus*	Senegal	Savanna	15	9 h 23 m 52 s
Kayan	*P. t. verus*	Senegal	Savanna	16	4 h 6 m 58 s
Bafing	*P. t. verus*	Mali	Savanna	5	0 h 29 m 40 s
Issa Valley	*P. t. schweinfurthii*	Tanzania	Savanna	25	7 h 14 m 1 s
Goualougo	*P. t. troglodytes*	Congo	Dense Forest	16	7 h 10 m 50 s
Campo Ma'an	*P. t. troglodytes*	Cameroon	Dense Forest	6	0 h 54 m 0 s
Mt. Cristal	*P. t. troglodytes*	Gabon	Dense Forest	11	3 h 11 m 25 s
La Belgique	*P. t. troglodytes*	Cameroon	Dense Forest	15	6 h 37 m 55 s
Wonga‐Wongué	*P. t. troglodytes*	Gabon	Dense Forest	11	5 h 32 m 3 s
Korup	*P. t. ellioti*	Cameroon	Dense Forest	31	7 h 1 m 52 s
Ganga	*P. t. ellioti*	Cameroon	Forest Mosaic	17	7 h 58 m 48 s

*Note:* This table includes information about the 10 chimpanzee communities analyzed in Boesch et al. (2020), whose data were also used in this study, as well as the Ganga community unique to this study. Information includes the community name, chimpanzee subspecies, community country location, habitat type, and the number of individuals used in Boesch et al. (2020) analyses. Location and habitat type information was gathered from Kalan et al. (2020) and Lindshield et al. (2021).

### 2.3 Data Preparation

#### 2.3.1 Technique Identification

We programmatically quantified all possible 2‐, 3‐, 4‐, 5‐, 6‐, and 7‐ sequential unit combinations of behavioral elements in chimpanzee videos taken at Ganga using Python, as previous studies of chimpanzee termite fishing across multiple communities have indicated that termite fishing techniques tend to consist of 3–7 elements (Boesch et al. 2020; Sanz and Morgan 2011). Technical combinations were deemed functional termite fishing techniques if they contained a probing element followed by an extraction element (Table S3). These were used to determine the overall repertoire of termite fishing techniques in the Ganga community, as well as the repertoires of each individual Ganga chimpanzee.

#### 2.3.2 Quantitative Ethnographic Preparation

We used ENA to quantify and visualize variation in termite fishing among individuals within and among communities considered in this study. ENA is a quantitative ethnographic technique that identifies and visualizes patterns of associations across themes in complex, large‐scale coded data (Shaffer 2017). ENA draws associations between cognitive/behavioral elements displayed in sequence by using their presence and absence within the recent temporal context in coded data. This framework aligns with the interpretations in this study of termite fishing techniques constructed from flexible combinations of individual behavioral elements (Barany et al. 2021; Andres‐Bray et al. 2023; Shaffer 2017). ENA is performed by representing behavioral elements as present or absent in coded rows and building matrices of associations using a moving stanza window, which connects relevant behavioral elements present in a single line of data to codes applied to prior lines within the recent temporal context (Shaffer 2017; Barany et al. 2021). These matrices of associations are then used to create visualized epistemic networks showing the patterns of association between behavioral elements for designated individuals or groups (Shaffer 2017). Shaffer et al. (2016) provide a useful tutorial for the ENA process for further information. Researchers use these epistemic networks to visually and statistically compare groups (units) to examine how they combine behavioral elements when the combinations of these elements are of particular interest (Shaffer 2017). For each unit group, network graphs quantify and visualize the associations (lines) between cognitive/behavioral elements (nodes) that represent how frequently two elements are used together in sequence. Although predominantly used in the learning sciences, an interdisciplinary field focusing on how people learn across a variety of contexts, researchers have recently begun using ENA in a variety of cross‐disciplinary contexts to analyze (a) operative performance of surgery trainees in simulated procedures (Ruis et al. 2018); (b) gaze coordination during collaborative work (Andrist et al. 2015); (c) health care team communication (Sullivan et al. 2018), and now (d) in examinations of patterns of chimpanzee tool use (Andres‐Bray et al. 2023).

Data were formatted so that each row represented the presence/absence of a behavioral element in a particular time frame to prepare them for ENA and to align with the data from Boesch et al. (2020) (Table S2). For ENA, rare behavioral elements from Boesch et al. (2020) where non‐applicable values, representing a situation where behavior was not observed but its absence could be influenced by context, accounted for greater than 90% of sequences were removed from the data set. All other blank and NA cells in the Boesch et al. (2020) data were changed to absences, as ENA cannot contend with missing data. For behavioral elements that are frequently used among chimpanzees within individual communities, this approach would have minimal impact on the associations drawn by the model for these elements. For rare behavioral elements, this approach avoids artificially inflating their connections with other elements by assuming they are present in a chimpanzee's repertoire when they may not be. Although this conservative approach may underestimate cultural similarity between these communities by distorting the absence of connections, it allows for interpretation of behavioral similarity by focusing on patterns of behaviors known to be present in these communities.

### 2.4 Statistics

#### 2.4.1 Individual Variation at Ganga

We used rarefaction curves with 1000 bootstrap replicates to identify chimpanzees at Ganga with sufficient observation time to reliably identify the diversity of their entire termite fishing technical repertoires. This process randomly samples from the observations of each of the 17 chimpanzees seen at Ganga to determine how many new behavioral elements are likely to be observed with each added observation. When the curves plateau, this signifies that additional observations of the corresponding chimpanzee are not contributing new behavioral elements, and thus, we can assume we have captured the majority of the behavioral variation demonstrated from that chimpanzee, though it is important to note that rare elements that were never seen used by a given chimpanzee may still be missed. This analysis identified 9 subjects out of the total 17 observed at Ganga that had sufficient observation time to capture the majority of their behavioral variation (Adult: *n* = 4; Juvenile: *n* = 3; Infant: *n* = 2) (Figure S2). Data from these nine subjects were used to analyze termite fishing techniques in the Ganga community. We used Fisher's Exact test to identify significant differences in use for each technique between individual chimpanzees (Fisher 1922). Fisher's exact test is useful with sparse data, which in this study resulted from a large number of techniques being unused by each chimpanzee (Fisher 1922). Finally, we conducted a cluster analysis to visualize similarity in technical repertoires among these nine Ganga chimpanzees.

#### 2.4.2 Community Comparison

When performing analyses comparing termite fishing variation between chimpanzee communities, data from all 17 chimpanzees observed at Ganga were included, because although some chimpanzees were not observed on camera enough to reliably measure the total variation of their termite fishing behavior, they serve together to approximate the total variation of termite fishing behavior present within the Ganga community. We again used rarefaction curves with 1000 bootstrap replicates at the community level to confirm each community had sufficient observed sequences of chimpanzee termite fishing to capture their full termite fishing cultural repertoires. These curves demonstrated that all 10 communities from Boesch et al. (2020) and the Ganga community both had sufficient observations to capture the majority of their behavioral variation (Figure S3). Information regarding the number of chimpanzees included in this analysis and the total observation time for each community can be found in Table 1.

To examine how chimpanzees at Ganga compare to other chimpanzee communities, we used Sørensen's Similarity Index on all paired combinations of communities included in this study (Sorensen 1948). Sørensen's Similarity Index is a useful metric to compare behavioral similarity between communities as it provides greater weight to shared elements compared to other similarity indices, and is calculated as:

$${\text{Sørensen}}_{i,j}(S)=2\;\times \;N_{\text{shared}}/(2\;\times \;N_{\text{shared}}+N_{\text{only}i}+N_{\text{only}j})$$

This calculation accounts for behavioral elements present in at least one of the two communities calculated, and provides a value from 0 to 1, where 0 would indicate no shared behavioral elements and 1 would indicate all behavioral elements were shared between both communities.

As Sørensen's Similarity Index looks at behavioral similarity between communities through the presence and absence of specific elements, we also used ENA to explore similarities in how these elements are combined within the same period of termite fishing using the ENA Web Tool (version 1.7.0) (Marquart et al. 2018). Two ENA models were generated to compare chimpanzee community repertoires using all behavioral elements analyzed by Boesch et al. (2020), one looking at termite fishing at underground nests and one looking at termite fishing at aerial nests. The aerial model contained 16 elements (see Table S2 for definitions):

- General: Rigid stick, soft stick, sit, and lean
- Tool making: Fray bite and fray pull
- Tool use: Probe 1 h, Probe 2 h, shake side, oscillate, extract 1 h, extract 2 h, wrist help, forearm help, and sweep
- Non‐tool use: Scratch ground

The underground model included 21 elements (see Table S2 for definitions):

- General: Long stick, lay, lean, and sit
- Tool making: Reduce, fray bite, and fray pull
- Tool use: Tap ground, penetrate 1 h, penetrate 2 h, lip shake, elbow insert, probe 2 h, hand help, wrist help, eat termite from wrist, eat termite by moving head, head to stick, leave tool, and help tool
- Non‐tool use: Mop

The resulting positions of each community's unit mean and the 95% confidence interval boxes surrounding them serve as a measure of how similar they are in terms of their termite fishing behavioral repertoires and how they combine behavioral elements during termite fishing. Both the aerial model (SVD1: Pearson = 0.98, Spearman = 0.98; SVD2: Pearson = 0.98, Spearman = 0.98) and the underground model (SVD1: Pearson = 0.97, Spearman = 0.97; SVD2: Pearson = 0.98, Spearman = 0.97) had strong goodness of fit, suggesting these models accurately reflect associations within the data. Finally, the ENA Web Tool (version 1.7.0) also calculates non‐parametric Mann–Whitney *U* tests between all paired combinations of communities to determine if their unit mean positions are significantly different along both the horizontal and vertical axes. Significant differences along either axis would signify that the communities being compared have significantly different ways of combining behavioral elements to fish for termites that are potentially the result of a combination of social, environmental, and/or genetic differences.

## 3 Results

### 3.1 Individual Variation at Ganga

**Figure 1:**
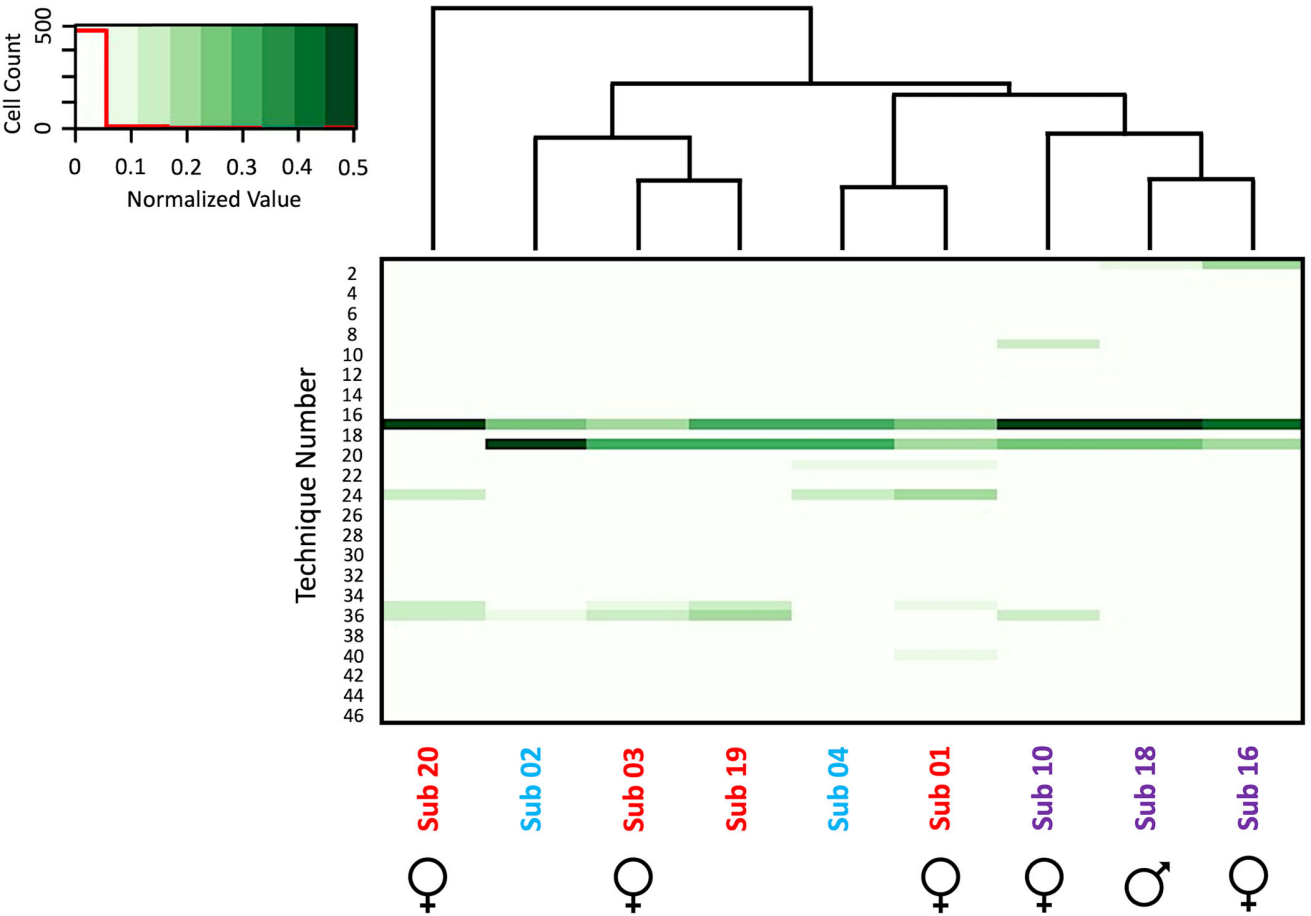
Heatmap with cluster analysis based on frequency of use of 46 techniques at Ganga. Frequencies of use were normalized by dividing by the highest frequency in the matrix to fit between 0 and 1. Technique number correspond to those in Table S3. Subjects are colored by age class (Adult: Red; Juveniles: Purple; Infants: Blue), and subject sex is identified where known.

We observed 46 distinct technical combinations of termite fishing behavioral elements used by chimpanzees at Ganga (Tables S3 and S4). Of the nine chimpanzees assessed for termite fishing variation within Ganga, individual repertoire size varied between 4 and 22 total techniques (*x̄* = 12.67, median = 13). The length of techniques employed by individual chimpanzees did not vary significantly with age class, though adults employed longer sequences of elements on average (Infants: *x̄* = 2.81 elements; Juveniles: *x̄* = 2.75 elements; adults: *x̄* = 3.05 elements). Fisher's exact test revealed 13 techniques with significantly different usage frequencies across 74 chimpanzee pairs (Table S5). Subject 18, a juvenile male, showed the greatest deviation in termite fishing repertoire from other Ganga chimpanzees, differing significantly in frequency of use across all 13 techniques. This is due in part to Subject 18's strong bias within their own repertoire for the prototypical “Basic Fish” and “Shake Fish” techniques, which involve the combinations probe‐extract and probe‐oscillate‐extract respectively. Finally, the cluster analysis identified high similarity between suspected mother‐offspring pairs (Sub 1/Sub 4 and Sub 3/Sub 2), as well as high similarity between the three juveniles in this study, which is consistent with known patterns of tool‐use transmission in chimpanzees (Figure 1) (Lamon et al. 2017). It is important to note that the mother/offspring relationship between these two pairs of chimpanzees is assumed based on video evidence of nursing, but given that this community is unhabituated and has only recently been observed via camera traps, the exact relationship between these chimpanzees is unknown and will be the subject of future studies. Subjects 1 and 4 had similar frequencies of use of four techniques: “Basic Fish” and “Shake Fish,” which have previously been described, as well as “Basic Wrist Help” and “Shake Wrist Help,” which are variations of these two techniques that replace the Extract element with the Wrist Help element by using the opposite wrist to guide the end of the tool from the mound to the subject's mouth. (Figure 1) (Table S4). Subjects 2 and 3 also have similar frequencies of use of the “Basic Fish” and “Shake Fish” techniques, as well as similar use of the “Shake Fish + Reshape” technique, which involves using the Reshape element to straighten the fibers of the tool's brush tip before performing “Shake Fish” (Figure 1) (Table S4). Finally, the three juveniles included in this analysis were clustered together based on similar frequency of use of the “Basic Fish” and “Shake Fish” techniques and similar use of the “Serial Basic Fish” technique, where chimpanzees repeat combinations of Probe‐Extract without bringing the tool to the mouth in a process that appears to be an extreme version of oscillating the tool (Figure 1) (Table S4).

### 3.2 Community Comparison

**Figure 2:**
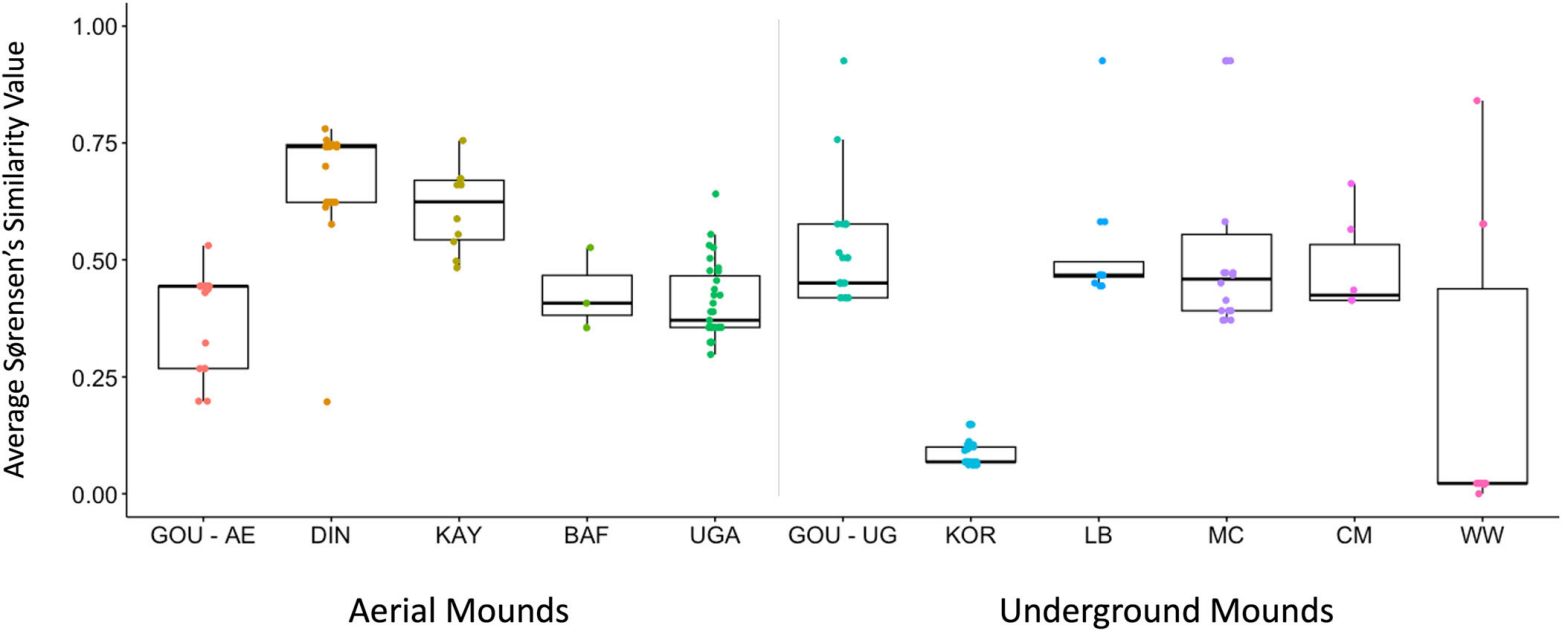
Average Sørensen's Similarity index between chimpanzees within each community and Ganga chimpanzees fishing at the same mound type (aerial or underground). This analysis exclusively includes behavioral elements assumed to be socially driven (Boesch et al. 2020). Horizontal lines within the boxes represent median values, the tops/bottoms of the boxes represent first and third quartile, vertical lines represent 2.5% and 97.5% quantiles, and circles represent individual chimpanzees. BAF, Bafing community; CM, Campo Ma'an community; DIN, Dindefelo community; GOU–UG, Goualougo community at underground mounds; GOU–AE, Goualougo community at aerial mounds; IV, Issa Valley community; KAY, Kayan community; KOR, Korup community; LB, La Belgique community; MC, Mt. Cristal community; WW, Wonga‐Wongue community.

Sørensen's Similarity Index (*S*) calculations were used to situate the behaviors used by Ganga chimpanzees to termite fish among other more well‐studied chimpanzee communities. Figure 2 shows the average *S* between all chimpanzees in each community observed by Boesch et al. (2020) with the members of all 17 chimpanzees Ganga chimpanzee community (see Table 1 for complete sample sizes and observation times). Ganga chimpanzees showed greatest similarity in behavioral elements used in termite fishing with chimpanzees at Dindefelo (*S* = 0.742) and Kayan (*S* = 0.624) at aerial termite mounds and chimpanzees at La Belgique (*S* = 0.467) and Mt Cristal (*S* = 0.459) at underground termite mounds. Further, Ganga chimpanzees demonstrated the least similarity overall in behavioral elements used in termite fishing with chimpanzees at Korup (*S* = 0.068) and Wonga‐Wongué (*S* = 0.022) at underground termite mounds (Figure 2).

**Figure 3:**
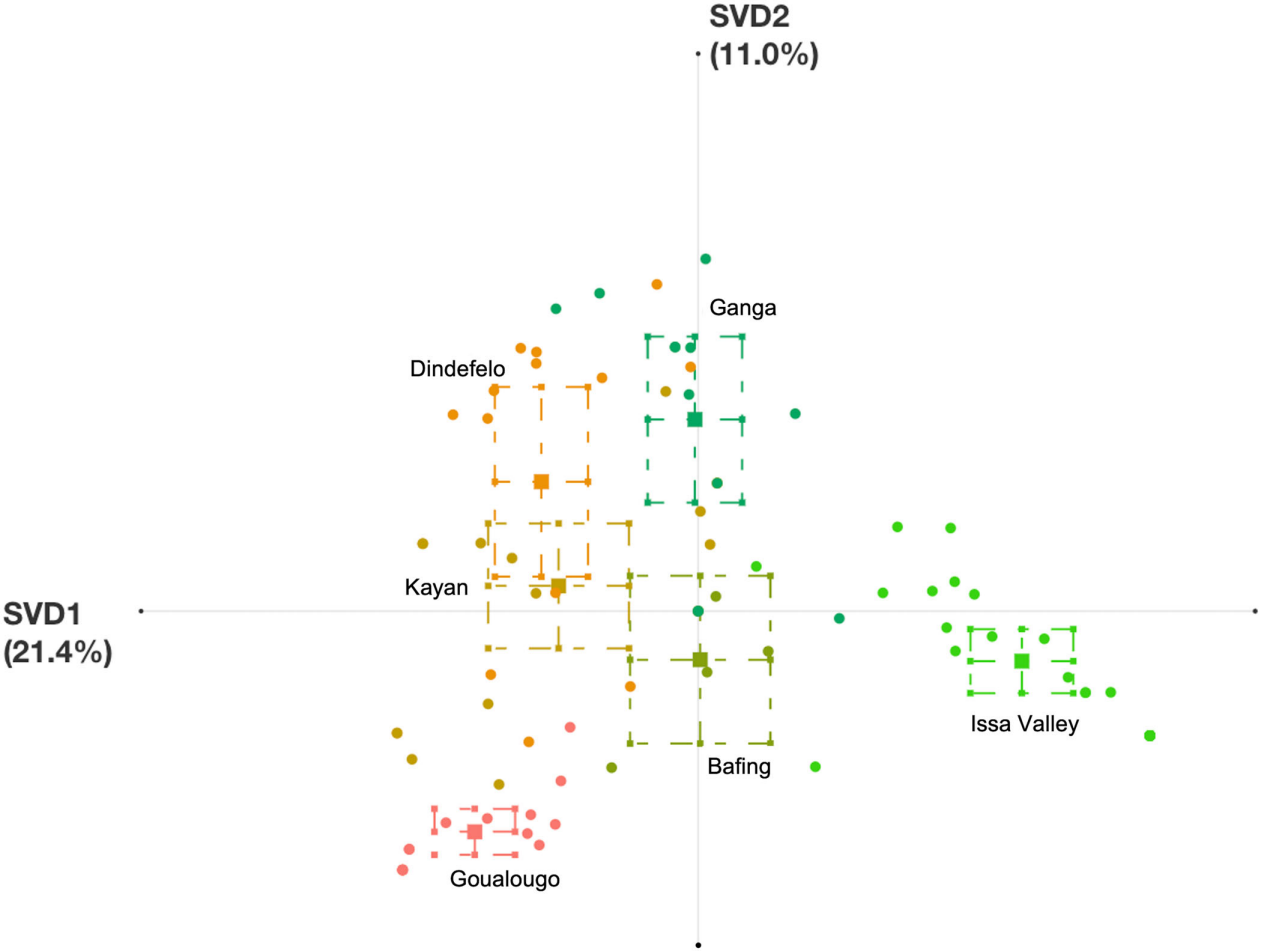
Epistemic network distribution of chimpanzees from six different communities performing termite fishing at aerial termite mounds. Squares represent unit means for each community, and circles represent individual chimpanzees from each community. Dotted boxes represent 95% confidence intervals along the horizontal (SVD1) and vertical (SVD2) axes. Variance explained by the horizontal axis = 21.4%, Variance explained by the vertical axis = 11.0%. Country locations of each community are as follows: Bafing, Mali; Dindefelo, Senegal; Goualougo, Congo; Issa Valley, Tanzania; Kayan, Senegal (Table 1).

**Figure 4:**
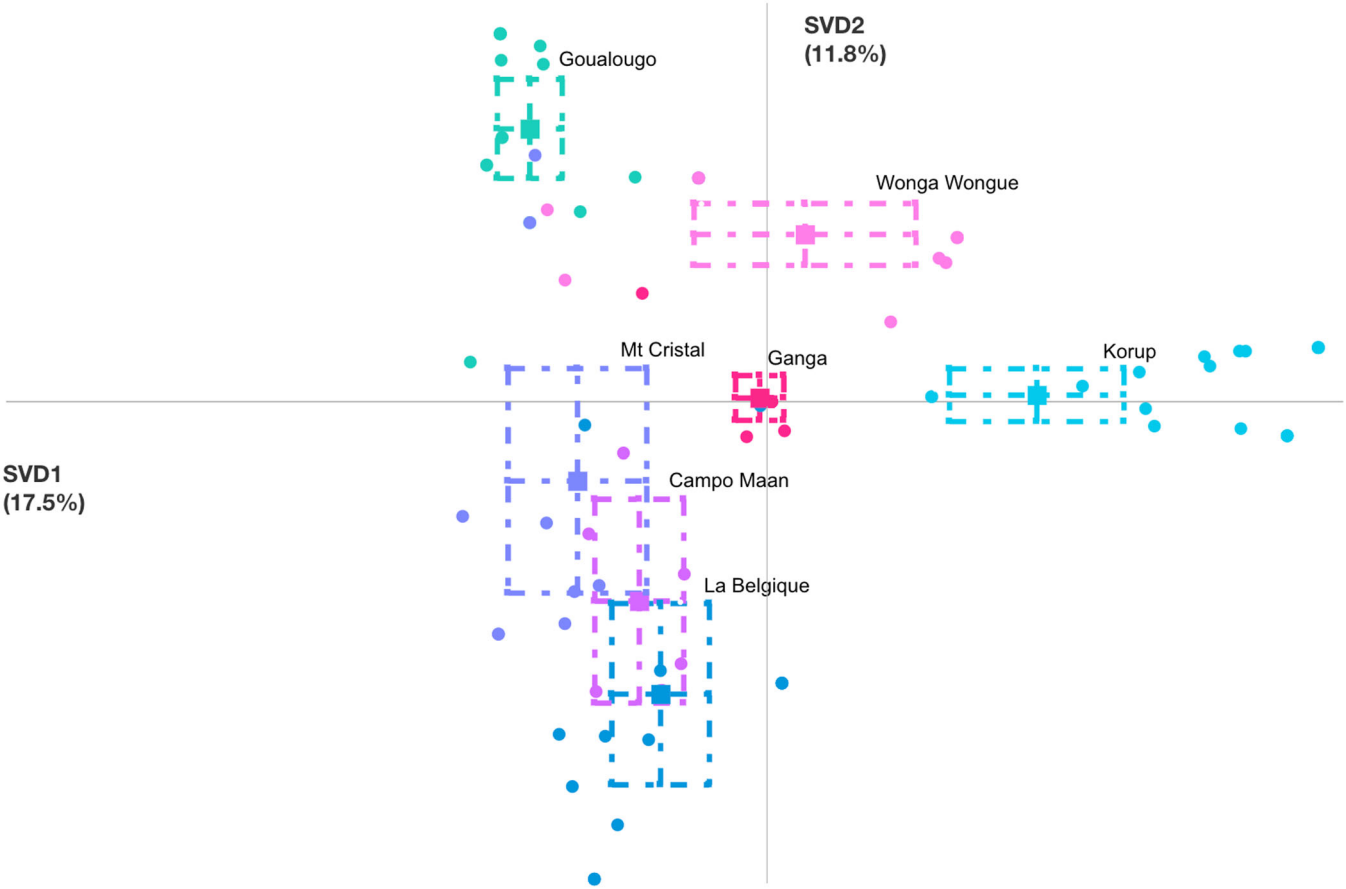
Epistemic network distribution of chimpanzees from seven different communities performing termite fishing at underground termite mounds. Squares represent unit means for each community, and circles represent individual chimpanzees from each community. Dotted boxes represent 95% confidence intervals along the horizontal (SVD1) and vertical (SVD2) axes. Variance explained by the horizontal axis = 17.5%, Variance explained by the vertical axis = 11.8%. Country locations of each community are as follows: Campo Ma'an, Cameroon; Goualougo, Congo; Korup, Cameroon; La Belgique, Cameroon; Mt. Cristal, Gabon; Wonga‐Wongué, Gabon (Table 1).

We generated two ENA models to further compare termite fishing between communities by exploring similarities in patterns of use of termite fishing behavioral elements within the same sequences at aerial mounds (Figure 3) and underground mounds (Figure 4). Confidence intervals in Figure 3 demonstrate that, at aerial termite mounds, the Goualougo, Issa Valley, and Ganga communities each have combinatorial patterns of termite fishing behavioral elements that differ from all other communities in this study. Individual chimpanzees in the Goualougo and Issa Valley in particular are strongly clustered around their respective unit means, suggesting strong community‐specific patterns of termite fishing within these two communities (Figure 3). Chimpanzees in the Ganga community, however, are interspersed within the model with savanna‐dwelling chimpanzees from the Dindefelo, Kayan, and Bafing communities in West Africa, which is consistent with the findings from the Sørensen Similarity Index (Figure 3). This suggests that chimpanzees among these four communities share some degree of behavioral similarity in their termite fishing behavior. This is confirmed by the results of the Mann–Whitney *U* tests, which demonstrate that the Ganga community is significantly different in terms of their patterns of termite fishing behavior from all communities except the Bafing community (horizontal axis) and the Dindefelo community (vertical axis) (Table 2).

**Table 2:** Results of Mann–Whitney *U* tests comparing ENA network similarity at aerial mounds.

Community	Goualougo	Dindefelo	Kayan	Bafing	Issa valley
A)^a^					
Dindefelo	136.00 (−0.39)	—	—	—	—
Kayan	143.00 (−0.36)	101.50 (0.03)	—	—	—
Bafing	70.00 (−1.00)***	3.00 (0.91)***	59.50 (−0.59)	—	—
Issa Valley	322.00 (−1.00)***	0.00 (1.00)***	345.00 (−1.00)***	114.00 (−0.98)***	—
Ganga	166.00 (−0.98)***	12.00 (0.86)***	135.00 (−0.50)*	23.00 (0.23)	3.00 (0.98)***
B)^b^					
Dindefelo	2.00 (0.98)***	—	—	—	—
Kayan	4.00 (0.96)***	58.50 (0.44)*	—	—	—
Bafing	1.00 (0.97)***	16.00 (0.54)	56.50 (−0.51)	—	—
Issa Valley	8.00 (0.95)***	65.00 (0.60)***	247.00 (−0.43)*	56.00 (0.03)	—
Ganga	0.00 (1.00)***	110.00 (−0.31)	36.00 (0.60)*	5.00 (0.83)*	21.00 (0.85)***

*Note:* Effect sizes are included in parentheses.

^a^ Compares communities along the horizontal axis SVD1.

^b^ Compares communities along the vertical axis SVD2.

* *p* < 0.05

** *p* < 0.01

*** *p* < 0.001.

**Figure 5:**
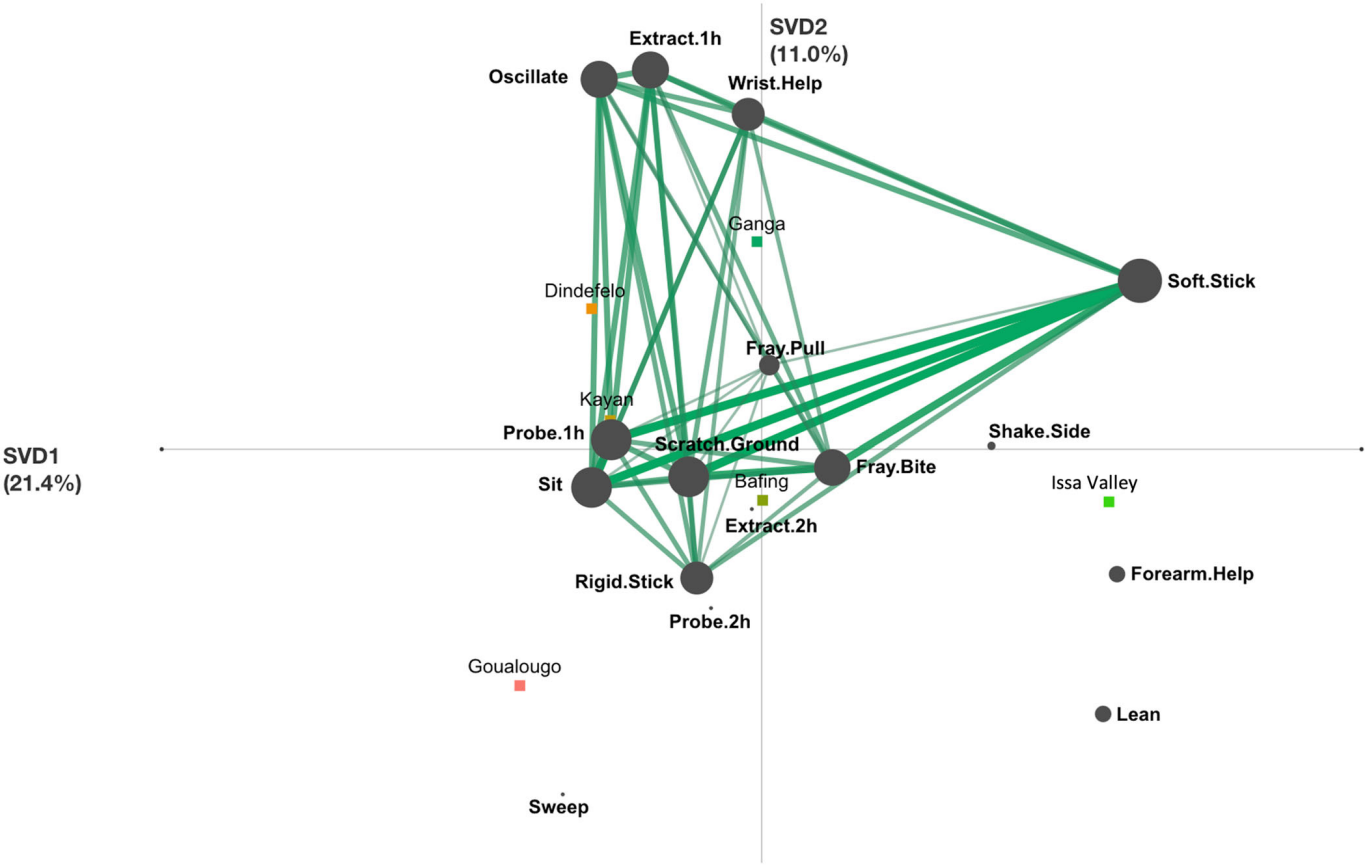
Epistemic network of termite fishing behavioral elements in the Ganga chimpanzee community at aerial termite mounds. This figure shows the connections between behavioral elements (circles) for Ganga chimpanzees. Stronger connections are represented by thicker lines. Variance explained by the horizontal axis = 21.4%, variance explained by the vertical axis = 11.0%. Squares represent unit means for each community.

The epistemic network for termite fishing at aerial mounds for the Ganga community is shown in Figure 5, and the networks for the other communities are included in Figures S4–S8. The Ganga network demonstrates particularly high associations among five putative socially transmitted elements: “Sit,” “Oscillate,” “Soft Stick,” “Wrist Help,” and “Scratch Ground” (Figure 5). This indicates that termite fishing at aerial mounds by Ganga chimpanzees is characterized by frequent combinations of fishing while sitting, using soft sticks to make fishing probes, scratching the mound in preparation of fishing, oscillating the inserted tool, and using the opposite wrist to help with tool extraction. By comparison, Dindefelo chimpanzees also frequently combine “Sit” and “Oscillate” but do not often use soft sticks, whereas chimpanzees at Kayan have strong connections with “Sit” and “Soft Stick” but do not oscillate the tool or use the opposite wrist during extraction (Figures S5 and S6). Bafing chimpanzees incorporate “Wrist Help” and “Scratch Ground” in their termite fishing at aerial mounds but more frequently use rigid sticks and lay on their sides (Figure S7). Finally, chimpanzees at Goualougo and Issa Valley were most different from other chimpanzee communities. Goualougo chimpanzees frequently combined “Sit” and “Rigid Stick” with the “Sweep” element that was largely unused in other communities (Figure S4), while Issa Valley chimpanzees were characterized by strong combinations of “Forearm Help,” which was also largely unused in other communities, with elements like “Soft Stick” and “Lean” (Figure S8).

The behavioral similarity between Ganga, Dindefelo, and Kayan may be primarily the result of ecological similarity. The large geographic distance between the *P. t. verus* communities of Dindefelo/Kayan and the *P. t. ellioti* community of Ganga would suggest a minimal impact of genetic similarity and/or social transmission of behavior. Dindefelo and Kayan are savanna‐dwelling communities whereas Ganga chimpanzees inhabit a complex ecotone of forest‐savanna matrix, and it may be that similarity in environmental factors between these communities (e.g., plant abundances, prey species/characteristics, soil quality) are contributing to shared patterns of termite fishing behavior, though more research would be needed to clarify the role of environment on chimpanzee termite fishing behavior in these communities.

In the underground model, confidence intervals also exhibited a high degree of difference between communities in terms of their community‐specific patterns of termite fishing (Figure 4). Individual chimpanzees from Goualougo, Korup, Wonga‐Wongué, and Ganga communities were strongly clustered near their respective unit means, suggesting strong community‐specific patterns of termite fishing (Figure 4). In contrast, the confidence intervals for the Campo Ma'an, Mt. Cristal, and La Belgique communities show a high degree of overlap, suggesting these three communities exhibit similar patterns of termite fishing at underground termite nests (Figure 4). The similarity between these three communities may be due to their relatively close geographic proximity in southern Cameroon/northern Gabon, which could suggest that genetic similarity, environmental similarity, and social transmission may all be working to some degree to facilitate similar patterns of termite fishing. Non‐parametric Mann–Whitney *U* tests in this model demonstrate that the Ganga community is significantly different in terms of their patterns of termite fishing behavior at underground termite nests for all communities except for Wonga‐Wongué (horizontal axis) and the Korup and Mt. Cristal communities (vertical axis) (Table 3), though it is important to note that the small effect sizes of most of these associations indicate that this similarity may be modest (Table 3). Members of the Korup community are strongly clustered together, indicating strong community‐specific combinations of termite fishing behaviors, though one member of the Korup community is located near the Ganga community mean, suggesting that individual chimpanzees fished for termites similarly to members of the Ganga community (Figure 4). Given that both communities live in populations known to exchange migrants (Mitchell et al. 2015), this could be evidence of genetic or social factors influencing behavioral similarity.

**Table 3:** Results of Mann–Whitney *U* tests comparing ENA network similarity at underground mounds.

Community	Goualougo	Korup	La Belgique	Mt cristal	Campo Ma'an	Wonga‐Wongue
A)^a^						
Korup	0.50 (1.00)***	—	—	—	—	—
La Belgique	20.00 (0.83)***	423.50 (−0.82)***	—	—	—	—
Mt Cristal	63.50 (0.28)	329.00 (−0.93)***	133.00 (−0.61)*	—	—	—
Campo Ma'an	9.00 (0.81)***	186.00 (−1.00)***	43.00 (0.04)	14.00 (0.58)	—	—
Wonga‐Wongue	7.50 (0.91)***	257.50 (−0.51)*	44.00 (0.47)*	15.00 (0.75)***	12.00 (0.64)*	—
Ganga	1.00 (0.99)***	305.00 (−0.64)***	43.50 (0.52)*	13.00 (0.80)***	3.00 (0.92)***	75.00 (−0.14)
B)^b^						
Korup	485.50 (−0.96)***	—	—	—	—	—
La Belgique	240.00 (−1.00)***	434.50 (−0.87)***	—	—	—	—
Mt Cristal	167.50 (−0.90)***	247.00 (−0.45)*	22.0 0 (0.73)***	—	—	—
Campo Ma'an	96.00 (−1.00)***	182.00 (−0.96)***	26.00 (0.42)	49.00 (−0.48)	—	—
Wonga‐Wongue	155.50 (−0.77)***	9.50 (0.94)***	0.00 (1.00)***	18.00 (0.70)**	0.00 (1.00)***	—
Ganga	191.00 (−0.99)***	219.00 (−0.18)	10.50 (0.88)***	37.00 (0.44)	0.00 (1.00)***	131.00 (−0.98)***

*Note:* Effect sizes are included in parentheses.

^a^ Compares communities along the horizontal axis SVD1.

^b^ Compares communities along the vertical axis SVD2.

* *p* < 0.05

** *p* < 0.01

*** *p* < 0.001.

**Figure 6:**
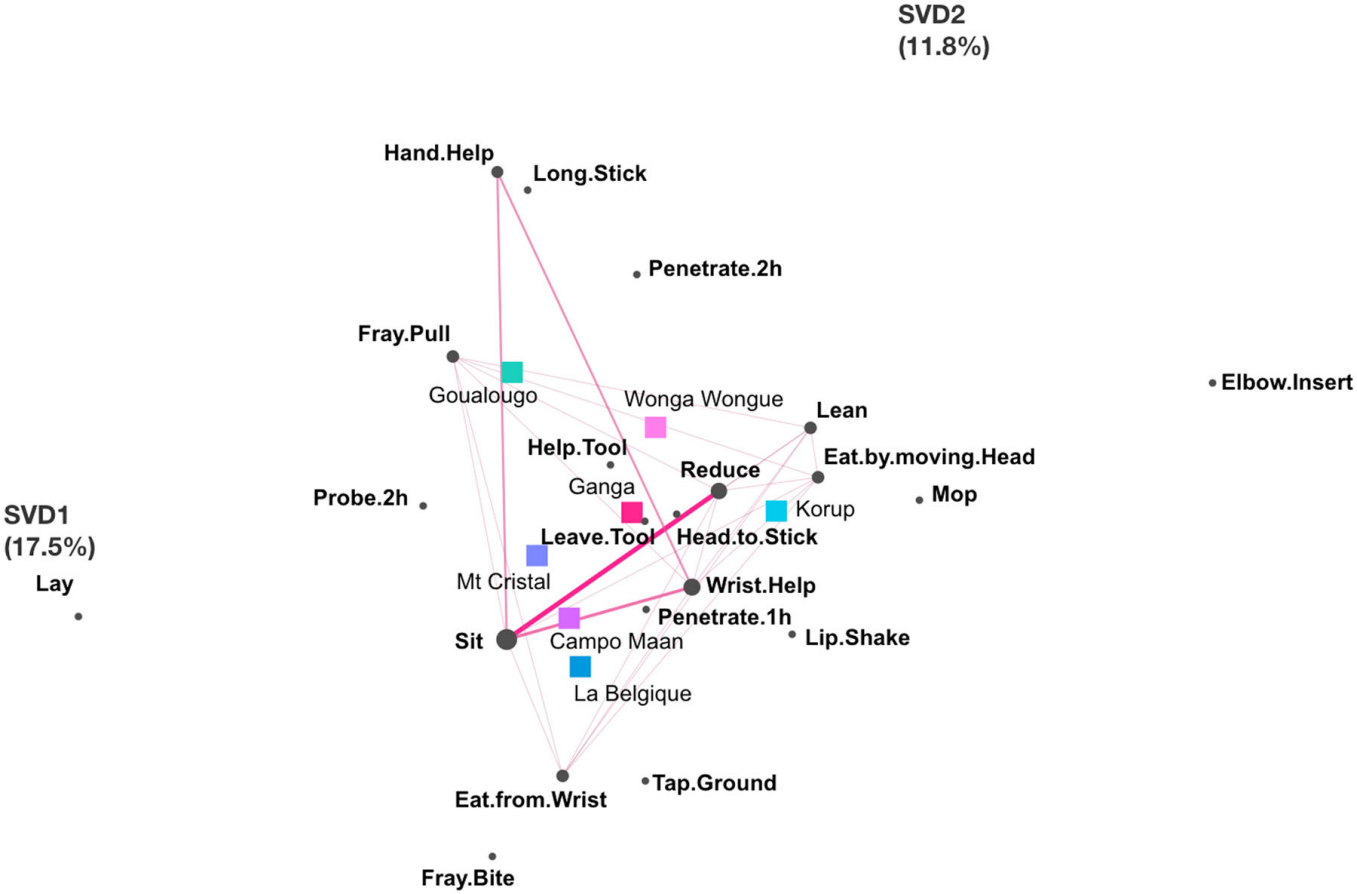
Epistemic network of termite fishing behavioral elements in the Ganga chimpanzee community at underground termite mounds. This figure shows the connections between behavioral elements (circles) for Ganga chimpanzees. Stronger connections are represented by thicker lines. Variance explained by the horizontal axis = 17.5%, variance explained by the vertical axis = 11.8%. Squares represent unit means for each community.

The epistemic network for termite fishing at underground mounds for the Ganga community is shown in Figure 6, and the networks for the other communities are included in Figures S9–S14. The Ganga network for termite fishing at underground termite nests shows very few strong connections (Figure 6). There is a strong association between “Sit” and “Reduce” and a strong triad of connections between “Sit,” “Hand Help,” and “Wrist Help,” indicating that termite fishing at underground termite nests by Ganga chimpanzees is characterized by fishing while sitting, removing parts of the tool to maintain tool function, and using the opposite hand/wrist to aid in extraction (Figure 6). The sitting position and the frequent use of the opposite limb for tool extraction are also used at aerial mounds, which may suggest that Ganga chimpanzees have a set of behavioral elements used during termite fishing that they employ even across different mound structures. Goualougo chimpanzees also frequently combine “Sit” and “Hand Help” while fishing at underground termite nests, however this is also combined with regular use of “Long Stick” and “Penetrate 2 h”, reflecting their community's unique sequential tool use to exploit termites living deep underground (Figure S9) (Sanz and Morgan 2007). In contrast, Korup chimpanzees have strong connections with the “Lean,” “Lip Shake,” “Eat by Moving Head,” and “Elbow Insert” elements, indicating a very different pattern of termite fishing behavior compared to Ganga chimpanzees (Figure S10). In contrast, the cluster formed by the La Belgique, Mt. Cristal, and Campo Ma'an communities all fish predominantly while sitting, and both the La Belgique and Mt. Cristal communities utilize the “Hand Help” element like members of the Ganga community, but chimpanzees in these three communities do not frequently utilize the “Reduce” or “Wrist Help” elements (Figures S11–S13). Finally, the Wonga‐Wongué chimpanzees frequently combine the “Long Stick” and “Penetrate 1 h” elements, showing they prepare for fishing by using long sticks to open up fishing holes in the underground termite nests (Figure S14). This community also incorporates the “Lay,” “Eating by Moving Head,” and “Elbow Insert” elements, demonstrating a similar method of termite fishing with the Korup community but using a different posture (Figure S14).

## 4 Discussion

### 4.1 Patterns of Termite Fishing Behavior in the Ganga Chimpanzee Community

In this study, we examined elemental variation of a widespread form of chimpanzee tool use behavior—termite fishing—in the unhabituated Ganga community, a group of the understudied *P. t. ellioti* chimpanzee population in central Cameroon. We identified 46 different combinations of 2‐to‐5 behavioral elements that constitute putative termite fishing techniques, consisting of some form of tool‐insertion element and some form of tool‐extraction element, and often incorporating various combinations of elements associated with maintaining the tool, preparing the mound, shaking the inserted tool, or assisting the tool towards the mouth for termite consumption. Although nearly all chimpanzees observed termite fishing in this community exhibited many of the elements that make up these techniques, no individual chimpanzee had a repertoire of more than 22 techniques and these individual repertoires feature unique combinations of the 46 techniques seen in the community at large. By clustering the chimpanzees at Ganga based on similarity of technique use, we were able to identify groups of similar termite fishing usage that to some degree mirrors known social groups captured on video. For example, suspected mother‐offspring pairs Subject1/Subject 4 and Subject 3/Subject 2 were grouped together in this analysis, as well as the three juvenile subjects in a third cluster, possibly the result of social transmission between chimpanzees and/or due to genetic similarities between individuals that may impact dexterity and propensity for certain prey (Langergraber et al. 2011). These results are consistent with the understood mechanism of tool‐use learning, which follows an initial vertical transmission from mother to offspring followed by secondary horizontal transmission among cohort‐mates (Lamon et al. 2017).

### 4.2 Situating Termite Fishing at Ganga Among Other Chimpanzee Communities

Several comparative analyses in this study identified other chimpanzee communities that utilize putatively socially driven behavioral elements to fish for termites similarly to the chimpanzees at Ganga. Overall, chimpanzees in the Ganga community appeared similar in several respects to chimpanzees in the Dindefelo, Kayan, and La Belgique communities. La Belgique is geographically close to Ganga relative to other chimpanzee communities in this study, and previous research in this region has shown that there are low levels of gene flow from the *P. t. troglodytes* population in southern Cameroon where La Belgique is located into the *P. t. ellioti* population in central Cameroon containing the Ganga community (Mitchell et al. 2015). Thus, it is possible that behavioral similarities in termite fishing between the La Belgique and Ganga communities are in part due to social influence via dispersal and/or genetic similarity, though the influence of both of these is likely low as the degree of migration is approximately one migrant from the *P. t. troglodytes* population in the south into the *P. t. ellioti* population in central Cameroon per generation (Mitchell et al. 2015). However, the Dindefelo and Kayan communities are located far from Ganga, and thus behavioral similarities between these two communities and chimpanzees at Ganga are more likely driven by environmental similarity, though more research would be needed to confirm this and to rule out the influences of other factors that impact behavioral similarity. Dindefelo and Kayan chimpanzees occupy savanna habitats in Senegal (Lindshield et al. 2021), whereas the chimpanzees at Ganga are found in a complex forest‐savanna ecotone in central Cameroon (Abwe et al. 2019). This suggests that there may be some environmental factors shared between these three communities that are contributing to similar behaviors in their termite fishing behaviors. The importance of the environment in shaping tool use similarity is further supported by the low behavioral similarity between chimpanzees at Ganga and Korup. There is considerable gene flow between west and central Cameroon (Mitchell et al. 2015), indicating opportunity for genetic and social factors to contribute to behavioral similarity. Of note is the one member of the Korup community placed near the Ganga community mean by the ENA, which could indicate a migrant from the ecotone population, but genetic data would be needed to confirm this. However, Korup is located in a dense rainforest habitat, which may be driving differences in termite fishing behaviors between Korup and Ganga (Lindshield et al. 2021).

The variation in similarity between chimpanzees at Ganga and chimpanzees in other communities indicates that there is unlikely a universal “optimal” termite fishing technique upon which all chimpanzee communities would converge. This is further supported by the lack of similarity between termite fishing behavior seen at Issa Valley in Tanzania relative to other savanna communities, highlighting the role that more fine‐scale ecological or genetic factors may play in shaping behavioral variation in chimpanzee termite fishing that could ultimately contribute to putatively cultural variation in tool use as the behaviors formed as a result of environmental and genetic factors begin to transmit socially. For example, differences in termite species characteristics may explain some variation in termite fishing behavior between chimpanzee communities. Several chimpanzee communities have been observed ignoring abundant termite species (e.g., *Odontotermes spp*.) potentially due to their taste or other species‐specific characteristics that affect their accessibility (Lesnik 2014; Pascual‐Garrido and Scheffrahn 2020; Sanz et al. 2014). Evidence suggests that Ganga chimpanzees are primarily predating upon an uncommon mound‐building termite species, *S. sphaerothorax*, which may contribute to termite fishing differences in this community, though more research on this termite species’ behaviors are necessary to explore this relationship (Andres‐Bray et al. 2024). The patterns of behaviors used in termite fishing by Ganga chimpanzees as a response to *S. sphaerothorax* characteristics could then potentially be spread to other nearby communities via dispersal and be maintained insofar as these patterns of behavior continue to be successful in other environmental contexts. Given the level of similarity found between Ganga chimpanzees and other distantly located and distantly related chimpanzees, added to the lack of similarity between Ganga chimpanzees and other *P. t. ellioti* in western Cameroon, elemental differences in termite fishing between communities appear to be due in part to the impact of ecological differences related to environment and/or prey characteristics.

### 4.3 Implications for Chimpanzee Culture

The results of this study provide further evidence of complex interactions between environmental factors and potential chimpanzee cultural behavior. It may be that cultural variation in termite fishing tool use begins as a behavioral adaptation to local environmental context, such as habitat type, prey species and behavior, and/or material availability (Koops et al. 2013). There is evidence that community‐level differences in tool material preference for probing tools are in part the result of material availability (Almeida‐Warren et al. 2017; McBeath and McGrew 1982; Pascual‐Garrido 2019). Chimpanzees have been shown to rely more strongly on existing knowledge rather than incorporate new behavioral elements to accomplish a goal (Gruber 2016), which could suggest that communities that have learned to use probing tools in particular ways may be more likely to continue using these patterns of behavior regardless of whether they are the most efficient way to accomplish a particular goal. Additionally, several studies have demonstrated that tool use behaviors are influenced by the behavior of the prey being targeted, suggesting that prey species and behavior may in part determine whether chimpanzees are able to predate upon them (Andres‐Bray et al. 2024; Phillips et al. 2023; Schöning et al. 2008; Sanz et al. 2014). Given that fine‐scale ecological characteristics may contribute to shaping cultural variation in tool use, greater ecological complexity may contribute to increased behavioral diversity and innovation, a pattern that has been observed in birds (LeFebvre et al. 2004; Sayol et al. 2016; Sol et al. 2005; Street et al. 2017).

More direct research is needed to pinpoint the specific ways that ecological characteristics contribute to chimpanzee cultural variation. As more chimpanzee communities have their putative cultural behaviors observed, it is important to consider how environmental factors contribute to observed cultural variation. Fine‐scaled ecological data on factors like prey behavior/nest characteristics, soil quality, soil humidity, mound hardness, vertical stratification, nutrients present in the soil, terrestrial herbaceous vegetation, climate variables, and more—collected in a similar way across chimpanzee communities—could help explain the environmental context driving these behaviors. Further, research has demonstrated that similarities in tool use behaviors between neighboring communities highly correlate with shared mitochondrial DNA variation, suggesting that shared ancestry predisposes chimpanzees to particular tool use behaviors (Langergraber et al. 2011). A comprehensive analysis combining fine‐scale ecological data with genetic data across neighboring chimpanzee communities would help clarify what aspects of behavioral similarity in insect‐directed tool use behaviors are associated with environmental influence and/or genetic factors, and what aspects are indicative of chimpanzee culture.

Finally, quantitative ethnographic methodologies like ENA from disciplines examining aspects of human cognition may provide new and beneficial avenues for exploring chimpanzee behavioral variation and the factors that influence it. Quantitative ethnography is a powerful analytical frame that facilitates quantitative assessment of observational/qualitative data, and ENA is especially useful for identifying differences in patterns of behaviors between contexts. In this regard, QE has potentially important applications in the study of the development of complex animal behavior and exploring behavioral differences between different environmental and social contexts, which highlights the potentially valuable contributions of interdisciplinary research to primatology.

## Author Contributions

**Tyler C. Andres‐Bray:** conceptualization (lead), data curation (lead), formal analysis (lead), methodology (lead), writing – original draft (lead), writing – review and editing (lead). **Ian Nichols:** methodology (equal), writing – original draft (supporting). **Tabitha Wilke:** methodology (supporting), writing – original draft (supporting), writing – review and editing (equal). **Macy Hafner:** methodology (supporting), writing – original draft (supporting), writing – review and editing (supporting). **Abigail Jordan:** methodology (supporting), writing – original draft (supporting). **Andrea Eysseric:** methodology (supporting), writing – original draft (supporting). **Vivianna Borzym:** methodology (supporting), writing – original draft (supporting). **Ekwoge E. Abwe:** funding acquisition (equal), investigation (equal), project administration (equal), writing – original draft (supporting). **Bethan Morgan:** funding acquisition (lead), writing–original draft (supporting). **Mary Katherine Gonder:** conceptualization (equal), data curation (supporting), funding acquisition (lead), methodology (supporting), project administration (lead), resources (lead), supervision (lead), writing – original draft (equal), writing – review and editing (supporting).

## Conflicts of Interest

The authors declare no conflicts of interest.

## Supporting information

Supporting information.

## Data Availability

This study incorporated data collected by Boesch et al. (2020) which is available at the following location: https://www.nature.com/articles/s41562-020-0890-1#Sec15. Data collected in this study from the Ganga chimpanzee community is available from the corresponding author upon reasonable request.
