# Supplementary material for: Patterns of Technical Variation in Chimpanzee Termite Fishing Behavior in Mbam and Djerem National Park, Cameroon
Source: Am J Primatol. 2025 Mar 2;87(3):e70014. doi: 10.1002/ajp.70014 (PMC11872189; doi:10.1002/ajp.70014)
Supplement: Supplementary file 1 — Supporting information. [file AJP-87-e70014-s001.docx]

***Supplemental Materials***

| 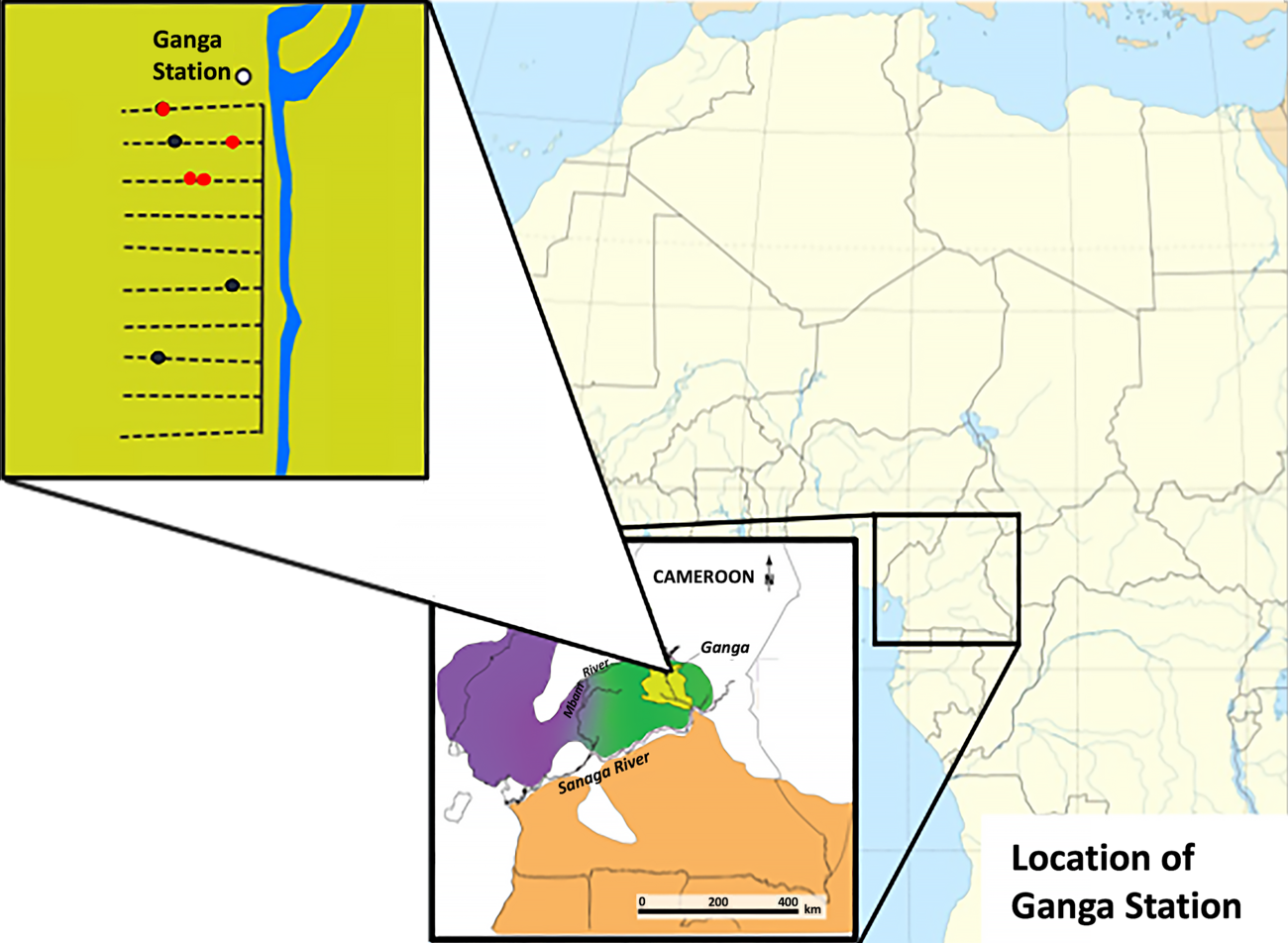 |
| --- |
| Figure S1. Location of termite mounds along transects near Ganga Research Station. Ganga Research Station is found in the northeastern part of MDNP, which is located in both the Adamawa and Centre regions of central Cameroon. Since 2016, monthly biomonitoring has been conducted along 10 2km-long transects that extend perpendicular to the Djerem river at 0.5 km intervals. The red and black dots represent termite mounds where camera traps were placed. Red dots are mounds where chimpanzees have been seen on camera termite fishing and black dots are mounds with no video evidence of chimpanzee termite fishing. Transects are numbered based on distance from Ganga Research Station. Purple, green, and orange areas in the map represent genetically distinct populations of chimpanzees from Mitchell et al. (2015): Two P. t. ellioti populations in a rainforest habitat (purple) and ecotone habitat (green), and one population of P. t. troglodytes (orange). Adapted from Andres-Bray et al., 2024. |

**Table S1**

*Placement Information for Each Camera Trap*

| Mound Name | Date Placed | Time Placed | Mound Type | Transect | Transect Location (m) | Latitude | Longitude | Habitat Type | Canopy |
| --- | --- | --- | --- | --- | --- | --- | --- | --- | --- |
| Mound A | 2/14/20 | 14:00 | Underground | 8 | 1750 | 5.99461 | 12.87311 | Secondary | Closed |
| Mound B | 2/16/20 | 12:45 | Underground | 3 | 825 | 5.97368 | 12.86533 | Secondary | Open |
| Mound C | 2/15/20 | 10:20 | Underground | 6 | 350 | 5.79604 | 12.86969 | Secondary | Open |
| Mound E | 2/16/20 | 12:30 | Aerial | 3 | 875 | 5.97367 | 12.86483 | Secondary | Open |
| Mound F | 2/17/20 | 10:10 | Aerial | 1 | 1415 | 5.98241 | 12.85952 | Secondary | Closed |
| Mound H | 2/17/20 | 9:50 | Aerial | 2 | 1200 | 5.97814 | 12.86216 | Gallery | Open |
| Mound I | 2/17/20 | 9:25 | Aerial | 2 | 650 | 5.97794 | 12.86716 | Secondary | Open |
| Mound J | 1/12/21 | 10:00 | Aerial | 3 | 750 | 5.97369 | 12.86601 | Secondary | Open |

*Note.* Each mound was identified by a unique letter. The date and time of each placement, mound type, transect, distance along transect, latitude/longitude, and habitat characteristics are listed.

**Table S2**

*Ethogram of Chimpanzee Behaviors*

| Behavioral Element | Boesch Element | Description |
| --- | --- | --- |
| Tool/Body Characteristics | | |
| Rigid Stick | Yes | Chimpanzee uses a stick does not bend when held in the hand or mouth |
| Soft Stick | Yes | Chimpanzee uses a stick that does bend when held in the hand or mouth |
| Long Stick | Yes | Stick is roughly as long as chimpanzee is tall (~1m) |
| Short Stick |  | Stick is shorter than 1m |
| Stand |  | Chimpanzee hindquarters are not in contact with the ground, at least one foot and one hand are on ground during fishing, or both feet and/or one hand are on ground during fishing |
| Sit | Yes | Chimpanzee hindquarters are on ground and is not using limbs to support body |
| Lean | Yes | Chimpanzee leans on the elbow of one arm while fishing with the opposite hand |
| Lay | Yes | Chimpanzee has one side of their body plus one shoulder touching the ground while fishing |
| Tool Making | | |
| Combine |  | Join/connect two or more objects to make tool to be held/manipulated as a single unit during use |
| Detach |  | Remove object (tool) from fixed connection to substrate or to another object |
| Reduce | Yes | Remove and discard part of an object, making it useful or a more efficient tool |
| Reshape |  | Chimpanzee pulls tool through hands/fingers to straighten fibers for inserting into termite nest |
| Fray - Bite | Yes | Bite with teeth the wood along the length of the distal extremity of the stick before inserting |
| Fray - Pull | Yes | Pull sidewards one end of the thin stick through teeth pressed together one or more times to produce a brushier end |
| Tool Use | | |
| Tap Ground | Yes | Tap the ground with the end of the thick stick held vertically in one hand at different locations above the mound before attempting to perforate |
| Penetrate 1h | Yes | Hold the thick stick with one hand and one foot to perforate the soil above the termite mound |
| Penetrate 2h | Yes | Hold the thick stick with both hands and one foot to forcefully push the stick into the soil above the termite mound to find a fishing spot |
| Probe 1h | Yes | Use only one hand to insert and extract the stick fishing for termites |
| Probe 2h | Yes | Use both hands alternatively or simultaneously to insert the stick to fish |
| Elbow Insert | Yes | Inserts or extracts probe near the elbow, likely to catch fallen termites when probe is removed. |
| Sidewrist | Yes | Place wrist/forearm of free hand under the thin stick when inserting and pulling out of the holes, the wrist gains an earth color with time |
| Failed Probe 1h |  | Use only one hand to attempt to insert the stick into the termite mound, but the stick is not inserted before reattempting |
| Failed Probe 2h |  | Use both hands to attempt to insert the stick into the termite mound, but the stick is not inserted before reattempting |
| Oscillate | Yes | Once the stick is inserted, the chimpanzee moves it up-and-down/in-and-out with the hand before pulling it out |
| Shake Side | Yes | The inserted stick is shaken sidewards with the hand during the fishing movement |
| Lip Shake | Yes | Chimpanzee uses lips to push or shake inserted probe before removing it with hands |
| Hand Help | Yes | Uses free hand under stick when extracted from the termite hole, so as to collect falling termites |
| Wrist help | Yes | Place wrist or side of wrist of free hand under the stick when extracted from the termite hole, so as to collect falling termites |
| Forearm help | Yes | The forearm upspine is following the movement of the stick to the mouth to catch termites that would release their grip |
| Extract 1h | Yes | The fishing hand alone pulls the stick out of the mound and brings the extremity of the stick with the termites directly in the mouth |
| Extract 2h | Yes | Both hands alternatively or simultaneously pull the stick out of the mound and brings the extremity of the stick with the termites directly in the mouth |
| Eat Termite from Tool |  | Chimpanzee eats termites directly from tool with mouth |
| Eat Termite from Wrist | Yes | Eat termites on the wrist and forearm of the supporting arm directly brought to the mouth. |
| Eat Termite by moving Head | Yes | Move the head forward to eat the termites directly from the stick with the lips |
| Head stick | Yes | Moves head towards stick to eat termites from stick supported by second hand |
| Gather |  | Use tool to gather termites from the surface of the mound |
| Reverse Orientation |  | Actively changes orientation of tool in relation to termite nest target (tool - switch but reverse the side of the tool used to probe the mound) |
| Block |  | Place object to prevent or impede movement or action of another object, fluid, or organism |
| Reach |  | Extend (without releasing) elongate object to touch or retrieve a goal object, out of reach or to maintain distance |
| Scratch |  | Move pointed object over bodily surface, repeatedly with pressure |
| Rub |  | Move unpointed object over bodily surface |
| Drop Tool |  | Chimpanzee releases tool on the surface of the mound or the ground and does not retrieve |
| Leave Tool |  | Chimpanzee releases the tool while inserted into the mound for less than 20 seconds |
| Leave Tool for 20 seconds | Yes | Chimpanzee releases the tool while inserted into the mound for at least 20 seconds |
| Help Tool | Yes | Chimpanzee assists in termite fishing with another chimpanzee or shares tool to sniff the end of the tool (usually adult to infant) |
| Transfer Tool |  | Chimpanzee gives tool to another chimpanzee (usually adult to infant) |
| Switch Tool |  | Chimpanzee switches tool between hands or mouth |
| Retrieve Tool |  | Chimpanzee picks up existing tool from the ground or nearby |
| Transport Tool |  | Chimpanzee travels along the ground with tool |
| Non-Tool Use | | |
| Aggress |  | Attack or threaten a conspecific |
| Appease |  | Make submissive gestures directed toward a dominant individual after aggression or in an attempt to prevent aggression |
| Snatch |  | Forcibly take item from another individual without consent |
| Groom Self |  | Use one or both hands to push hair back and pick at one's own exposed skin with nail of thumb or index finger |
| Groom Other |  | Use one or both hands to push hair back and pick at another individual's skin using thumb and index finger |
| Smell Object |  | Smells hands or tool - denote in body modifier column or comments the object being smelled |
| Scratch Ground | Yes | Scratches at an object/termite mound with finger and not tool |
| Rake Away Leaves |  | Uses hand to rake leaves or soil away from area where they are going to insert probe |
| Climb |  | Chimp locomotes above ground, up or down a trunk, branch, pole, or other vertical structure |
| Locomotion |  | Travel around on the ground |
| Play |  | Perform behaviors with conspecifics or alone that have no immediate use or purpose, often accompanied with particular "play" facial expressions and vocalizations |
| Rest |  | Remain immobile, sit or lying down |
| Sweep | Yes | Use the free hand to pull the termites away from the stick and bring them to the mouth with this hand |
| Pick |  | Pick termites up from surface of mound with hand, non-tool use termite capture for consumption |
| Mop Termite | Yes | Use the hair on the back of the hand to touch the termites on the ground for them to attach and they eat them. |
| Eat Termite not from Tool |  | Place insects into mouth without using tool, then bite and chew, wadge and swallow |
| Eat Termite Directly from Mound |  | Consume termites by putting mouth directly on the surface of the mound, then bite and chew, wadge and swallow. |
| Eat Termite from Debris |  | Consume termites by picking up unmodified sticks or plant matter from the surface of the mound that had termites on it |
| Eat Other |  | Place non-insects into mouth, bite and chew, wadge and swallow |
| Scan |  | Look up and/or around away from conspecifics in nearby area, theoretically observing for other organisms or reacting to sound |
| Watch |  | Focuses on actions of a conspecific |
| Other |  | Any behavior not described on this list. Description of behavior will be included in notes |
| Off Screen |  | Not visible on video and thus behavior is unknown |
|  | | |

*Note.* Video coding for this project involved 72 chimpanzee behaviors compiled from Boesch et al. 2020 and Nishida et al. 1999. Eight of these behaviors are characteristics of the tools being used as well as body positioning during fishing. Six of these behaviors are involved in tool making/maintenance, 35 were tool using behaviors, and 23 are non-tool use behaviors that can be used in termite fishing or social contexts and might also occur during the videos. The column “Boesch et al. 2020 Element” indicates whether the behavioral element in the ethogram for this study corresponded to an element from the Boesch et al. 2020 study.

**Table S3**

*Technical Combinations of Behavioral Elements Observed in Ganga Chimpanzees*

| ID | Technique Name | Element 1 | Element 2 | Element 3 | Element 4 | Element 5 | Count |
| --- | --- | --- | --- | --- | --- | --- | --- |
| 1 | Serial Basic Fish | Probe | Extract | Probe | Extract |  | 37 |
| 2 | Basic Fish + Fray Bite/Pull | Fray Bite | Fray Pull | Probe | Extract |  | 1 |
| 3 | Basic Fish + Fray Bite | Fray Bite | Probe | Extract |  |  | 8 |
| 4 | Shake Fish + Fray Bite | Fray Bite | Probe | Oscillate | Extract |  | 7 |
| 5 | Shake Wrist Help + Fray Bite | Fray Bite | Probe | Oscillate | Wrist Help |  | 1 |
| 6 | Basic Wrist Help + Fray Bite | Fray Bite | Probe | Wrist Help |  |  | 1 |
| 7 | Basic Fish + Fray Bite/Reduce | Fray Bite | Reduce | Probe | Extract |  | 1 |
| 8 | Shake Fish + Fray Bite/Reshape | Fray Bite | Reduce | Probe | Oscillate | Extract | 2 |
| 9 | Basic Fish + Fray Bite/Reshape | Fray Bite | Reshape | Probe | Extract |  | 1 |
| 10 | Shake Fish + Fray Bite/Reshape | Fray Bite | Reshape | Probe | Oscillate | Extract | 1 |
| 11 | Basic Wrist Help + Fray Bite/Reshape | Fray Bite | Reshape | Probe | Wrist Help |  | 1 |
| 12 | Basic Fish + Fray Pull | Fray Pull | Probe | Extract |  |  | 3 |
| 13 | Shake Fish + Fray Pull | Fray Pull | Probe | Oscillate | Extract |  | 9 |
| 14 | Shake Wrist Help + Fray Pull | Fray Pull | Probe | Oscillate | Wrist Help |  | 5 |
| 15 | Basic Wrist Help + Fray Pull | Fray Pull | Probe | Wrist Help |  |  | 2 |
| 16 | Basic Fish + Fray Pull/Reduce | Fray Pull | Reduce | Probe | Extract |  | 1 |
| 17 | Basic Fish | Probe | Extract |  |  |  | 297 |
| 18 | Basic Forearm Help | Probe | Forearm Help |  |  |  | 1 |
| 19 | Shake Fish | Probe | Oscillate | Extract |  |  | 261 |
| 20 | Shake Forearm Help | Probe | Oscillate | Forearm Help |  |  | 1 |
| 21 | Shake Wrist Help | Probe | Oscillate | Wrist Help |  |  | 47 |
| 22 | Side Shake Fish | Probe | Shake Side | Extract |  |  | 7 |
| 23 | Side Shake Wrist Help | Probe | Shake Side | Wrist Help |  |  | 2 |
| 24 | Basic Wrist Help | Probe | Wrist Help |  |  |  | 68 |
| 25 | Basic Fish + Reduce/Fray Bite | Reduce | Fray Bite | Probe | Extract |  | 1 |
| 26 | Shake Fish + Fray Bite/Reduce | Reduce | Fray Bite | Probe | Oscillate | Extract | 1 |
| 27 | Shake Fish + Fray Pull/Reduce | Reduce | Fray Pull | Probe | Oscillate | Extract | 1 |
| 28 | Basic Wrist Help + Reduce/Fray Pull | Reduce | Fray Pull | Probe | Wrist Help |  | 2 |
| 29 | Basic Fish + Reduce | Reduce | Probe | Extract |  |  | 2 |
| 30 | Shake Fish + Reduce | Reduce | Probe | Oscillate | Extract |  | 9 |
| 31 | Shake Wrist Help + Reduce | Reduce | Probe | Oscillate | Wrist Help |  | 1 |
| 32 | Basic Wrist Help + Reduce | Reduce | Probe | Wrist Help |  |  | 1 |
| 33 | Basic Fish + Fray Pull/Reshape | Reshape | Fray Pull | Probe | Extract |  | 1 |
| 34 | Shake Fish + Reshape/Fray Pull | Reshape | Fray Pull | Probe | Oscillate | Extract | 1 |
| 35 | Basic Fish + Reshape | Reshape | Probe | Extract |  |  | 52 |
| 36 | Shake Fish + Reshape | Reshape | Probe | Oscillate | Extract |  | 55 |
| 37 | Shake Forearm Help + Reshape | Reshape | Probe | Oscillate | Forearm Help |  | 1 |
| 38 | Shake Wrist Help + Reshape | Reshape | Probe | Oscillate | Wrist Help |  | 27 |
| 39 | Side Shake Fish + Reshape | Reshape | Probe | Shake Side | Extract |  | 1 |
| 40 | Basic Wrist Help + Reshape | Reshape | Probe | Wrist Help |  |  | 22 |
| 41 | Shake Wrist Help + Reshape/Scratch Ground | Reshape | Scratch Ground | Probe | Oscillate | Wrist Help | 1 |
| 42 | Basic Fish + Scratch Ground | Scratch Ground | Probe | Extract |  |  | 3 |
| 43 | Shake Fish + Scratch Ground | Scratch Ground | Probe | Oscillate | Extract |  | 4 |
| 44 | Shake Wrist Help + Scratch Ground | Scratch Ground | Probe | Oscillate | Wrist Help |  | 1 |
| 45 | Basic Wrist Help + Scratch Ground | Scratch Ground | Probe | Wrist Help |  |  | 1 |
| 46 | Basic Fish + Scratch Ground/Reshape | Scratch Ground | Reshape | Probe | Extract |  | 1 |

*Note.* Elements are shown in sequential order from 1 to 5, along with the total number of times each combination has been observed in use and the names given to each technique.

| 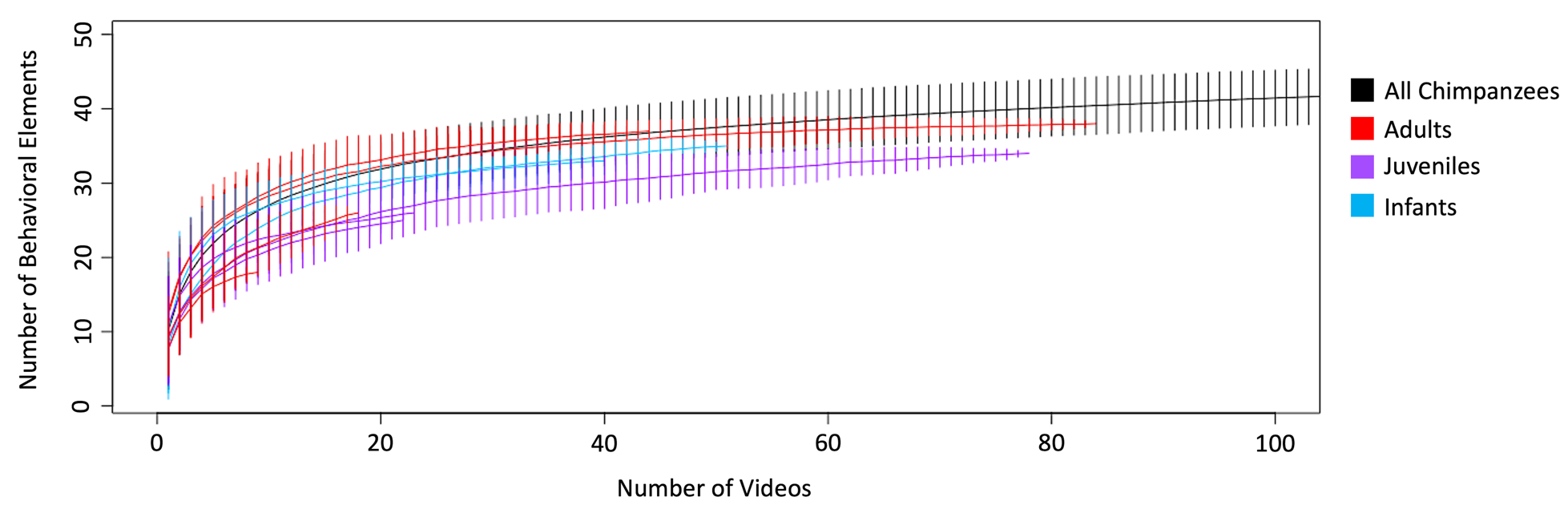 |
| --- |
| Figure S2. Rarefaction curve of behavioral elements with 1000 permutations comparing all chimpanzees with at least 9 videos of data. This figure shows that for all 9 subjects included, the total number of behavioral elements observed begins to level off once all of that subjects’ videos are included. This suggests that each of these subjects has sufficient data to capture the majority of their behavioral variation. Subjects are color coded by age class (Adult: *n* = 4; Juvenile: *n* = 3; Infant: *n* = 2). |

| 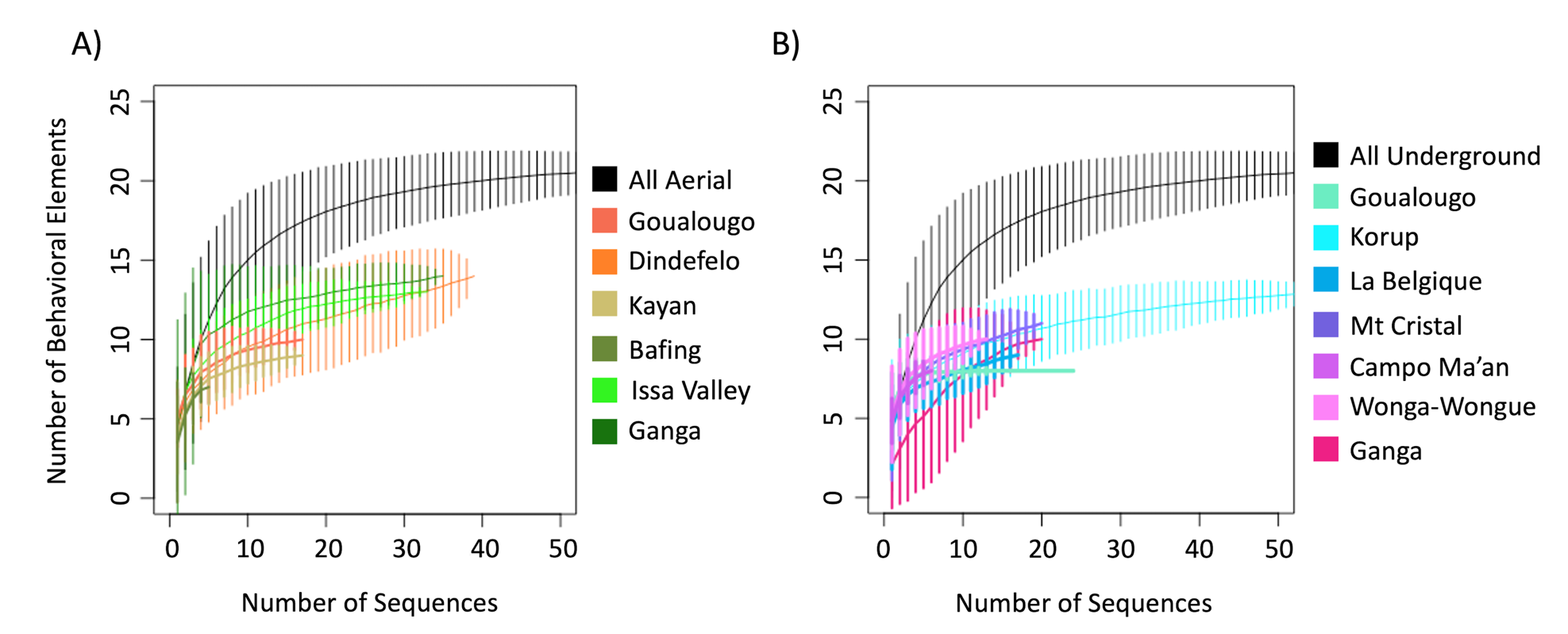 |
| --- |
| Figure S3: Rarefaction curve of behavioral elements with 1000 permutations comparing all chimpanzee communities fishing at A) aerial mounds and B) underground mounds. This figure shows that for all communities in this study, the total number of behavioral elements observed begins to level off once all of that community’s recorded sequences are included. This suggests that each of these communities has sufficient data to capture the majority of their behavioral variation. |

Table S4

*Frequency of use of each technique across all 17 subjects*

| ID | Technique | Sub01 | Sub02 | Sub03 | Sub04 | Sub05 | Sub06 | Sub08 | Sub09 | Sub10 |
| --- | --- | --- | --- | --- | --- | --- | --- | --- | --- | --- |
| 1 | Serial Basic Fish | 0 | 2 | 0 | 3 | 0 | 0 | 0 | 0 | 0 |
| 2 | Basic Fish + Fray Bite/Pull | 0 | 0 | 1 | 0 | 0 | 0 | 0 | 0 | 0 |
| 3 | Basic Fish + Fray Bite | 3 | 0 | 0 | 1 | 0 | 0 | 0 | 0 | 0 |
| 4 | Shake Fish + Fray Bite | 0 | 5 | 1 | 0 | 0 | 0 | 0 | 0 | 0 |
| 5 | Shake Wrist Help + Fray Bite | 0 | 0 | 1 | 0 | 0 | 0 | 0 | 0 | 0 |
| 6 | Basic Wrist Help + Fray Bite | 1 | 0 | 0 | 0 | 0 | 0 | 0 | 0 | 0 |
| 7 | Basic Fish + Fray Bite/Reduce | 0 | 0 | 1 | 0 | 0 | 0 | 0 | 0 | 0 |
| 8 | Shake Fish + Fray Bite/Reduce | 0 | 0 | 1 | 0 | 0 | 0 | 0 | 0 | 0 |
| 9 | Basic Fish + Fray Bite/Reshape | 0 | 0 | 0 | 0 | 0 | 0 | 0 | 0 | 1 |
| 10 | Shake Fish + Fray Bite/Reshape | 0 | 1 | 0 | 0 | 0 | 0 | 0 | 0 | 0 |
| 11 | Basic Wrist Help + Fray Bite/Reshape | 0 | 0 | 1 | 0 | 0 | 0 | 0 | 0 | 0 |
| 12 | Basic Fish + Fray Pull | 1 | 0 | 2 | 0 | 0 | 0 | 0 | 0 | 0 |
| 13 | Shake Fish + Fray Pull | 2 | 0 | 5 | 0 | 0 | 0 | 0 | 0 | 0 |
| 14 | Shake Wrist Help + Fray Pull | 0 | 0 | 5 | 0 | 0 | 0 | 0 | 0 | 0 |
| 15 | Basic Wrist Help + Fray Pull | 1 | 0 | 1 | 0 | 0 | 0 | 0 | 0 | 0 |
| 16 | Basic Fish + Fray Pull/Reduce | 0 | 0 | 1 | 0 | 0 | 0 | 0 | 0 | 0 |
| 17 | Basic Fish | 58 | 25 | 23 | 29 | 0 | 0 | 0 | 1 | 4 |
| 18 | Basic Forearm Help | 0 | 0 | 0 | 1 | 0 | 0 | 0 | 0 | 0 |
| 19 | Shake Fish | 42 | 56 | 37 | 28 | 0 | 0 | 0 | 0 | 2 |
| 20 | Shake Forearm Help | 0 | 0 | 1 | 0 | 0 | 0 | 0 | 0 | 0 |
| 21 | Shake Wrist Help | 22 | 1 | 6 | 10 | 0 | 0 | 0 | 0 | 0 |
| 22 | Side Shake Fish | 2 | 0 | 2 | 1 | 0 | 0 | 0 | 0 | 0 |
| 23 | Side Shake Wrist Help | 1 | 0 | 0 | 0 | 0 | 0 | 0 | 0 | 0 |
| 24 | Basic Wrist Help | 46 | 1 | 4 | 16 | 0 | 0 | 0 | 0 | 0 |
| 25 | Basic Fish + Reduce/Fray Bite | 1 | 0 | 0 | 0 | 0 | 0 | 0 | 0 | 0 |
| 26 | Shake Fish + Reduce/Fray Bite | 0 | 0 | 0 | 0 | 0 | 0 | 1 | 0 | 0 |
| 27 | Shake Fish + Fray Pull/Reduce | 0 | 0 | 1 | 0 | 0 | 0 | 0 | 0 | 0 |
| 28 | Basic Wrist Help + Reduce/Fray Pull | 1 | 0 | 0 | 1 | 0 | 0 | 0 | 0 | 0 |
| 29 | Basic Fish + Reduce | 2 | 0 | 0 | 0 | 0 | 0 | 0 | 0 | 0 |
| 30 | Shake Fish + Reduce | 0 | 5 | 1 | 1 | 0 | 0 | 0 | 0 | 0 |
| 31 | Shake Wrist Help + Reduce | 1 | 0 | 0 | 0 | 0 | 0 | 0 | 0 | 0 |
| 32 | Basic Wrist Help + Reduce | 1 | 0 | 0 | 0 | 0 | 0 | 0 | 0 | 0 |
| 33 | Basic Fish + Fray Pull/Reshape | 0 | 0 | 1 | 0 | 0 | 0 | 0 | 0 | 0 |
| 34 | Shake Fish + Reshape/Fray Pull | 0 | 0 | 1 | 0 | 0 | 0 | 0 | 0 | 0 |
| 35 | Basic Fish + Reshape | 18 | 3 | 9 | 3 | 0 | 0 | 0 | 0 | 0 |
| 36 | Shake Fish + Reshape | 8 | 9 | 18 | 2 | 0 | 0 | 0 | 0 | 1 |
| 37 | Shake Forearm Help + Reshape | 0 | 1 | 0 | 0 | 0 | 0 | 0 | 0 | 0 |
| 38 | Shake Wrist Help + Reshape | 13 | 1 | 5 | 2 | 0 | 0 | 0 | 0 | 0 |
| 39 | Side Shake Fish + Reshape | 0 | 0 | 0 | 0 | 0 | 0 | 0 | 0 | 0 |
| 40 | Basic Wrist Help + Reshape | 19 | 1 | 1 | 1 | 0 | 0 | 0 | 0 | 0 |
| 41 | Shake Wrist Help + Reshape/Scratch Ground | 0 | 0 | 0 | 0 | 0 | 0 | 0 | 0 | 0 |
| 42 | Basic Fish + Scratch Ground | 1 | 0 | 0 | 0 | 0 | 0 | 0 | 0 | 0 |
| 43 | Shake Fish + Scratch Ground | 0 | 0 | 2 | 0 | 0 | 0 | 0 | 0 | 0 |
| 44 | Shake Wrist Help + Scratch Ground | 0 | 0 | 0 | 0 | 0 | 0 | 0 | 0 | 0 |
| 45 | Basic Wrist Help + Scratch Ground | 1 | 0 | 0 | 0 | 0 | 0 | 0 | 0 | 0 |
| 46 | Basic Fish + Scratch Ground/Reshape | 0 | 0 | 0 | 0 | 0 | 0 | 0 | 0 | 0 |
|  | Total Techniques | 22 | 13 | 26 | 14 | 0 | 0 | 1 | 1 | 4 |

| ID | Technique | Sub16 | Sub17 | Sub18 | Sub19 | Sub20 | Sub21 | Sub23 | Sub30 |
| --- | --- | --- | --- | --- | --- | --- | --- | --- | --- |
| 1 | Serial Basic Fish | 12 | 0 | 19 | 1 | 0 | 0 | 0 | 0 |
| 2 | Basic Fish + Fray Bite/Pull | 0 | 0 | 0 | 0 | 0 | 0 | 0 | 0 |
| 3 | Basic Fish + Fray Bite | 2 | 0 | 2 | 0 | 0 | 0 | 0 | 0 |
| 4 | Shake Fish + Fray Bite | 0 | 0 | 0 | 1 | 0 | 0 | 0 | 0 |
| 5 | Shake Wrist Help + Fray Bite | 0 | 0 | 0 | 0 | 0 | 0 | 0 | 0 |
| 6 | Basic Wrist Help + Fray Bite | 0 | 0 | 0 | 0 | 0 | 0 | 0 | 0 |
| 7 | Basic Fish + Fray Bite/Reduce | 0 | 0 | 0 | 0 | 0 | 0 | 0 | 0 |
| 8 | Shake Fish + Fray Bite/Reduce | 0 | 0 | 0 | 0 | 0 | 0 | 0 | 0 |
| 9 | Basic Fish + Fray Bite/Reshape | 0 | 0 | 0 | 0 | 0 | 0 | 0 | 0 |
| 10 | Shake Fish + Fray Bite/Reshape | 0 | 0 | 0 | 0 | 0 | 0 | 0 | 0 |
| 11 | Basic Wrist Help + Fray Bite/Reshape | 0 | 0 | 0 | 0 | 0 | 0 | 0 | 0 |
| 12 | Basic Fish + Fray Pull | 0 | 0 | 0 | 0 | 0 | 0 | 0 | 0 |
| 13 | Shake Fish + Fray Pull | 0 | 0 | 2 | 0 | 0 | 0 | 0 | 0 |
| 14 | Shake Wrist Help + Fray Pull | 0 | 0 | 0 | 0 | 0 | 0 | 0 | 0 |
| 15 | Basic Wrist Help + Fray Pull | 0 | 0 | 0 | 0 | 0 | 0 | 0 | 0 |
| 16 | Basic Fish + Fray Pull/Reduce | 0 | 0 | 0 | 0 | 0 | 0 | 0 | 0 |
| 17 | Basic Fish | 24 | 0 | 119 | 11 | 3 | 0 | 0 | 0 |
| 18 | Basic Forearm Help | 0 | 0 | 0 | 0 | 0 | 0 | 0 | 0 |
| 19 | Shake Fish | 12 | 0 | 72 | 11 | 0 | 1 | 0 | 0 |
| 20 | Shake Forearm Help | 0 | 0 | 0 | 0 | 0 | 0 | 0 | 0 |
| 21 | Shake Wrist Help | 0 | 0 | 8 | 0 | 0 | 0 | 0 | 0 |
| 22 | Side Shake Fish | 0 | 0 | 2 | 0 | 0 | 0 | 0 | 0 |
| 23 | Side Shake Wrist Help | 0 | 0 | 1 | 0 | 0 | 0 | 0 | 0 |
| 24 | Basic Wrist Help | 0 | 0 | 4 | 0 | 1 | 0 | 0 | 0 |
| 25 | Basic Fish + Reduce/Fray Bite | 0 | 0 | 0 | 0 | 0 | 0 | 0 | 0 |
| 26 | Shake Fish + Reduce/Fray Bite | 0 | 0 | 0 | 0 | 0 | 0 | 0 | 0 |
| 27 | Shake Fish + Fray Pull/Reduce | 0 | 0 | 0 | 0 | 0 | 0 | 0 | 0 |
| 28 | Basic Wrist Help + Reduce/Fray Pull | 0 | 0 | 0 | 0 | 0 | 0 | 0 | 0 |
| 29 | Basic Fish + Reduce | 0 | 0 | 0 | 0 | 0 | 0 | 0 | 0 |
| 30 | Shake Fish + Reduce | 2 | 0 | 0 | 0 | 0 | 0 | 0 | 0 |
| 31 | Shake Wrist Help + Reduce | 0 | 0 | 0 | 0 | 0 | 0 | 0 | 0 |
| 32 | Basic Wrist Help + Reduce | 0 | 0 | 0 | 0 | 0 | 0 | 0 | 0 |
| 33 | Basic Fish + Fray Pull/Reshape | 0 | 0 | 0 | 0 | 0 | 0 | 0 | 0 |
| 34 | Shake Fish + Reshape/Fray Pull | 0 | 0 | 0 | 0 | 0 | 0 | 0 | 0 |
| 35 | Basic Fish + Reshape | 1 | 0 | 11 | 6 | 1 | 0 | 0 | 0 |
| 36 | Shake Fish + Reshape | 0 | 0 | 9 | 7 | 1 | 0 | 0 | 0 |
| 37 | Shake Forearm Help + Reshape | 0 | 0 | 0 | 0 | 0 | 0 | 0 | 0 |
| 38 | Shake Wrist Help + Reshape | 0 | 0 | 6 | 0 | 0 | 0 | 0 | 0 |
| 39 | Side Shake Fish + Reshape | 0 | 0 | 1 | 0 | 0 | 0 | 0 | 0 |
| 40 | Basic Wrist Help + Reshape | 0 | 0 | 0 | 0 | 0 | 0 | 0 | 0 |
| 41 | Shake Wrist Help + Reshape/Scratch Ground | 0 | 0 | 1 | 0 | 0 | 0 | 0 | 0 |
| 42 | Basic Fish + Scratch Ground | 0 | 0 | 2 | 0 | 0 | 0 | 0 | 0 |
| 43 | Shake Fish + Scratch Ground | 1 | 0 | 0 | 1 | 0 | 0 | 0 | 0 |
| 44 | Shake Wrist Help + Scratch Ground | 0 | 0 | 1 | 0 | 0 | 0 | 0 | 0 |
| 45 | Basic Wrist Help + Scratch Ground | 0 | 0 | 0 | 0 | 0 | 0 | 0 | 0 |
| 46 | Basic Fish + Scratch Ground/Reshape | 0 | 0 | 0 | 1 | 0 | 0 | 0 | 0 |
|  | Total Techniques | 7 | 0 | 16 | 8 | 4 | 1 | 0 | 0 |

*Note:* Full repertoire size is listed at the bottom of the table for each chimpanzee.

Table S5. Significant results of Fisher’s Exact Test performing pairwise comparisons between subjects based on frequency of use of each identified technique in the Ganga community.

| ID | Technique | Comparison | P-Value |
| --- | --- | --- | --- |
| 1 | Serial Basic Fish | Sub01 vs Sub04 | 0.023321 |
| 1 | Serial Basic Fish | Sub01 vs Sub16 | 4.02E-10 |
| 1 | Serial Basic Fish | Sub01 vs Sub18 | 3.13E-06 |
| 1 | Serial Basic Fish | Sub02 vs Sub16 | 3.08E-05 |
| 1 | Serial Basic Fish | Sub02 vs Sub18 | 0.046907 |
| 1 | Serial Basic Fish | Sub03 vs Sub16 | 1.38E-07 |
| 1 | Serial Basic Fish | Sub03 vs Sub18 | 0.000635 |
| 1 | Serial Basic Fish | Sub04 vs Sub16 | 0.000268 |
| 1 | Serial Basic Fish | Sub16 vs Sub18 | 0.002178 |
| 1 | Serial Basic Fish | Sub16 vs Sub19 | 0.006589 |
| 4 | Shake Fish + Fray Bite | Sub01 vs Sub02 | 0.002767 |
| 4 | Shake Fish + Fray Bite | Sub02 vs Sub18 | 0.002248 |
| 9 | Basic Fish + Fray Bite/Reshape | Sub01 vs Sub10 | 0.031621 |
| 9 | Basic Fish + Fray Bite/Reshape | Sub10 vs Sub18 | 0.029851 |
| 13 | Shake Fish + Fray Pull | Sub03 vs Sub18 | 0.045754 |
| 14 | Shake Wrist Help + Fray Pull | Sub01 vs Sub03 | 0.005005 |
| 14 | Shake Wrist Help + Fray Pull | Sub03 vs Sub18 | 0.004114 |
| 17 | Basic Fish | Sub01 vs Sub16 | 0.003746 |
| 17 | Basic Fish | Sub01 vs Sub18 | 2.44E-07 |
| 17 | Basic Fish | Sub02 vs Sub16 | 0.006099 |
| 17 | Basic Fish | Sub02 vs Sub18 | 2.48E-05 |
| 17 | Basic Fish | Sub03 vs Sub04 | 0.038767 |
| 17 | Basic Fish | Sub03 vs Sub10 | 0.044839 |
| 17 | Basic Fish | Sub03 vs Sub16 | 0.000315 |
| 17 | Basic Fish | Sub03 vs Sub18 | 1.9E-08 |
| 17 | Basic Fish | Sub04 vs Sub18 | 0.005654 |
| 19 | Shake Fish | Sub01 vs Sub02 | 2.74E-10 |
| 19 | Shake Fish | Sub01 vs Sub03 | 0.016747 |
| 19 | Shake Fish | Sub01 vs Sub04 | 0.026139 |
| 19 | Shake Fish | Sub01 vs Sub18 | 0.00552 |
| 19 | Shake Fish | Sub02 vs Sub03 | 0.000538 |
| 19 | Shake Fish | Sub02 vs Sub04 | 0.001193 |
| 19 | Shake Fish | Sub02 vs Sub16 | 0.000681 |
| 19 | Shake Fish | Sub02 vs Sub18 | 4.36E-05 |
| 19 | Shake Fish | Sub02 vs Sub19 | 0.023966 |
| 19 | Shake Fish | Sub02 vs Sub20 | 0.028135 |
| 21 | Shake Wrist Help | Sub01 vs Sub02 | 0.002176 |
| 21 | Shake Wrist Help | Sub01 vs Sub16 | 0.018347 |
| 21 | Shake Wrist Help | Sub01 vs Sub18 | 0.007419 |
| 21 | Shake Wrist Help | Sub02 vs Sub04 | 0.003464 |
| 21 | Shake Wrist Help | Sub04 vs Sub16 | 0.014763 |
| 21 | Shake Wrist Help | Sub04 vs Sub18 | 0.01213 |
| 24 | Basic Wrist Help | Sub01 vs Sub02 | 2.69E-07 |
| 24 | Basic Wrist Help | Sub01 vs Sub03 | 4.66E-06 |
| 24 | Basic Wrist Help | Sub01 vs Sub16 | 8.76E-05 |
| 24 | Basic Wrist Help | Sub01 vs Sub18 | 8.6E-12 |
| 24 | Basic Wrist Help | Sub01 vs Sub19 | 0.000806 |
| 24 | Basic Wrist Help | Sub02 vs Sub04 | 4.07E-05 |
| 24 | Basic Wrist Help | Sub03 vs Sub04 | 0.000614 |
| 24 | Basic Wrist Help | Sub04 vs Sub16 | 0.000691 |
| 24 | Basic Wrist Help | Sub04 vs Sub18 | 7.49E-07 |
| 24 | Basic Wrist Help | Sub04 vs Sub19 | 0.00591 |
| 30 | Shake Fish + Reduce | Sub01 vs Sub02 | 0.002767 |
| 30 | Shake Fish + Reduce | Sub01 vs Sub16 | 0.03212 |
| 30 | Shake Fish + Reduce | Sub02 vs Sub18 | 0.002248 |
| 30 | Shake Fish + Reduce | Sub16 vs Sub18 | 0.02912 |
| 35 | Basic Fish + Reshape | Sub02 vs Sub19 | 0.009942 |
| 35 | Basic Fish + Reshape | Sub04 vs Sub19 | 0.015465 |
| 35 | Basic Fish + Reshape | Sub16 vs Sub19 | 0.020221 |
| 35 | Basic Fish + Reshape | Sub18 vs Sub19 | 0.013966 |
| 36 | Shake Fish + Reshape | Sub01 vs Sub03 | 0.000384 |
| 36 | Shake Fish + Reshape | Sub01 vs Sub19 | 0.001531 |
| 36 | Shake Fish + Reshape | Sub02 vs Sub16 | 0.031265 |
| 36 | Shake Fish + Reshape | Sub03 vs Sub04 | 0.001624 |
| 36 | Shake Fish + Reshape | Sub03 vs Sub16 | 0.002003 |
| 36 | Shake Fish + Reshape | Sub03 vs Sub18 | 0.000447 |
| 36 | Shake Fish + Reshape | Sub04 vs Sub19 | 0.002111 |
| 36 | Shake Fish + Reshape | Sub16 vs Sub19 | 0.001624 |
| 36 | Shake Fish + Reshape | Sub18 vs Sub19 | 0.00179 |
| 40 | Basic Wrist Help + Reshape | Sub01 vs Sub02 | 0.006183 |
| 40 | Basic Wrist Help + Reshape | Sub01 vs Sub03 | 0.002872 |
| 40 | Basic Wrist Help + Reshape | Sub01 vs Sub04 | 0.018941 |
| 40 | Basic Wrist Help + Reshape | Sub01 vs Sub16 | 0.030152 |
| 40 | Basic Wrist Help + Reshape | Sub01 vs Sub18 | 7.4E-07 |

| 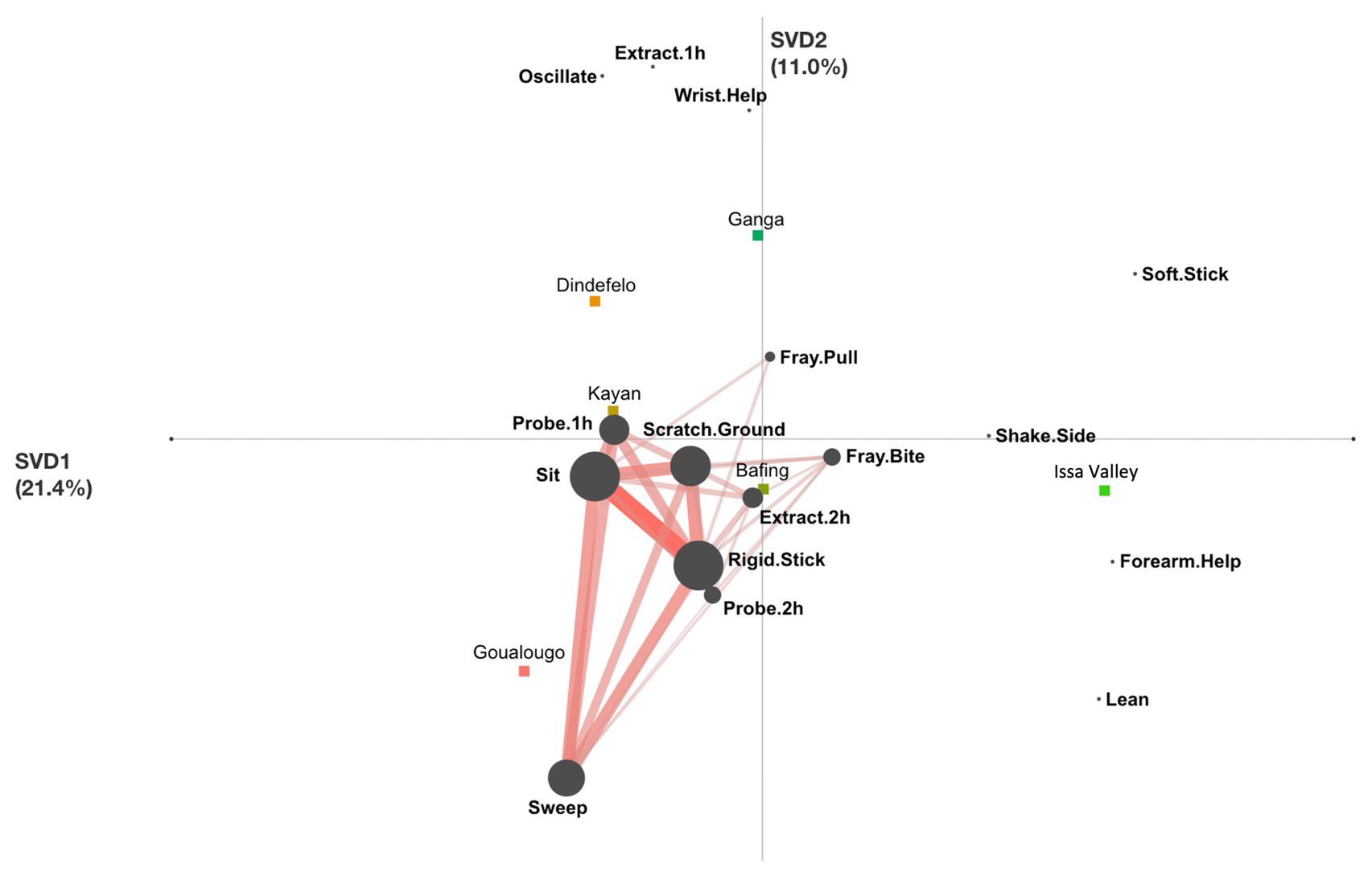 |
| --- |
| Figure S4. Epistemic network of termite fishing behavioral elements in the Goualougo chimpanzee community at aerial termite mounds. This figure shows the connections between behavioral elements (circles) for Goualougo chimpanzees. Stronger connections are represented by thicker lines. Variance explained by the x-dimension = 21.4%, Variance explained by the y-dimension=11.0%. Squares represent unit means for each community. |

| 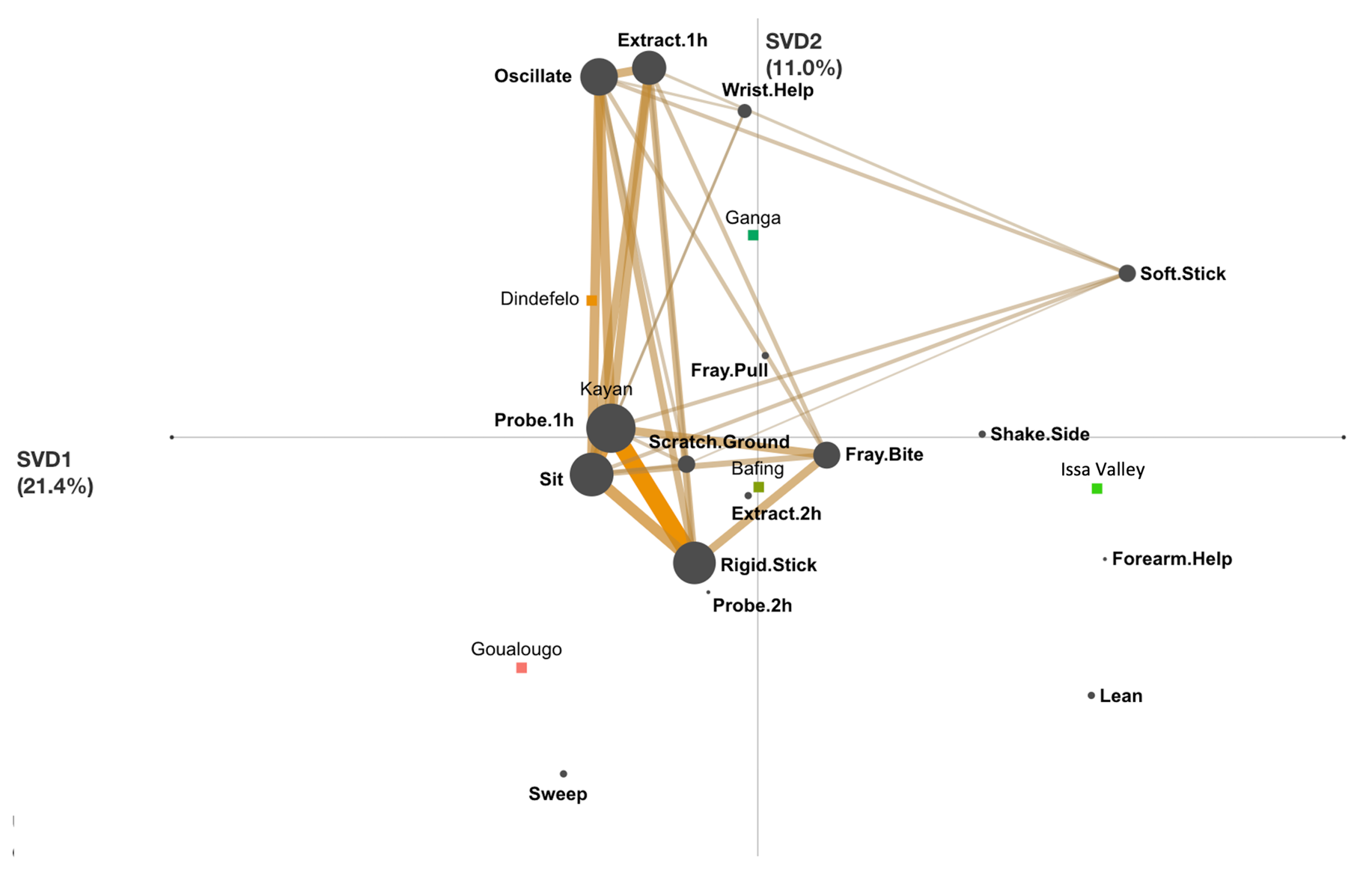 |
| --- |
| Figure S5. Epistemic network of termite fishing behavioral elements in the Dindefelo chimpanzee community at aerial termite mounds. This figure shows the connections between behavioral elements (circles) for Dindefelo chimpanzees. Stronger connections are represented by thicker lines. Variance explained by the x-dimension = 21.4%, Variance explained by the y-dimension=11.0%. Squares represent unit means for each community. |

| 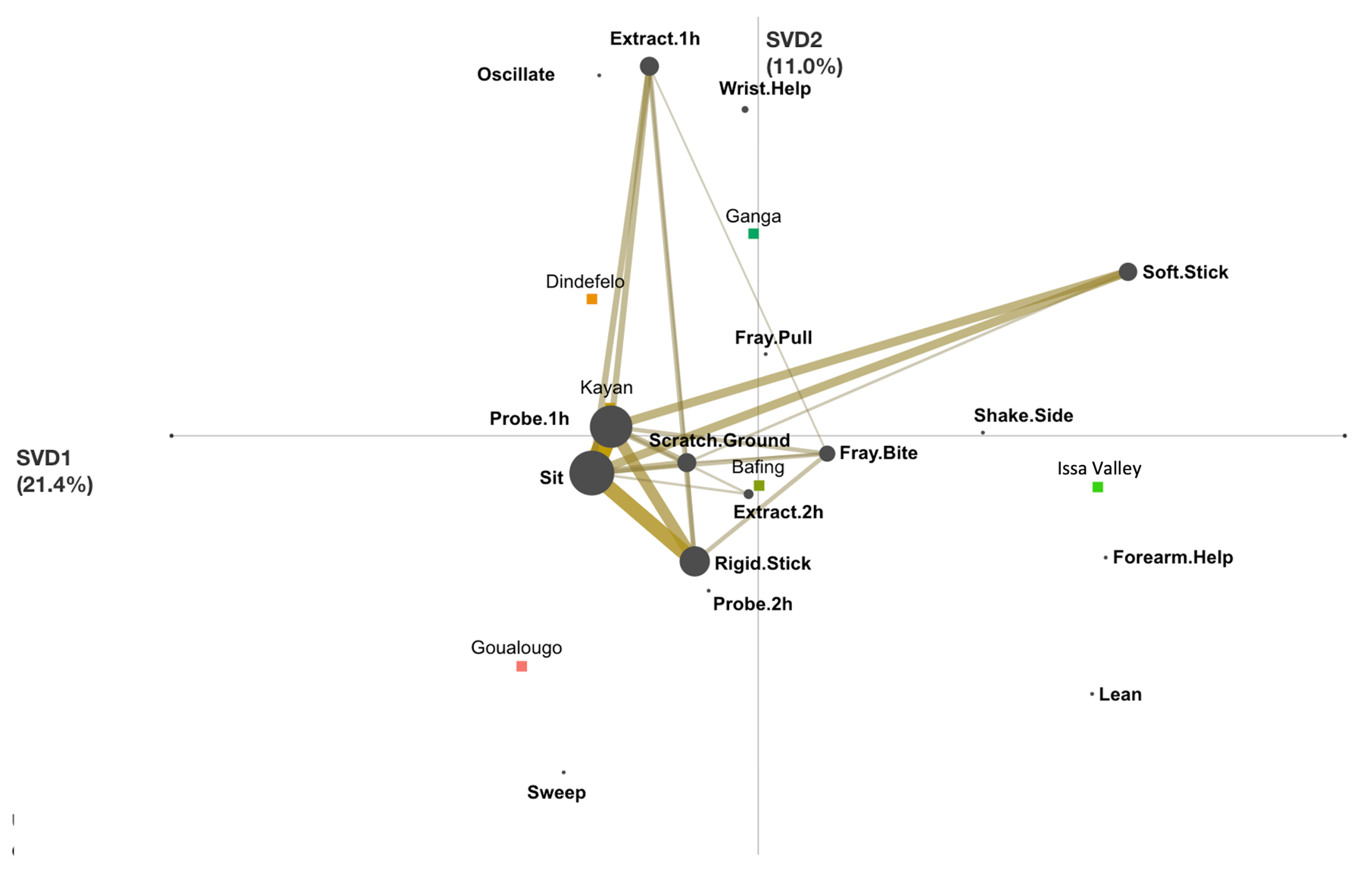 |
| --- |
| Figure S6. Epistemic network of termite fishing behavioral elements in the Kayan chimpanzee community at aerial termite mounds. This figure shows the connections between behavioral elements (circles) for Kayan chimpanzees. Stronger connections are represented by thicker lines. Variance explained by the x-dimension = 21.4%, Variance explained by the y-dimension=11.0%. Squares represent unit means for each community. |

| 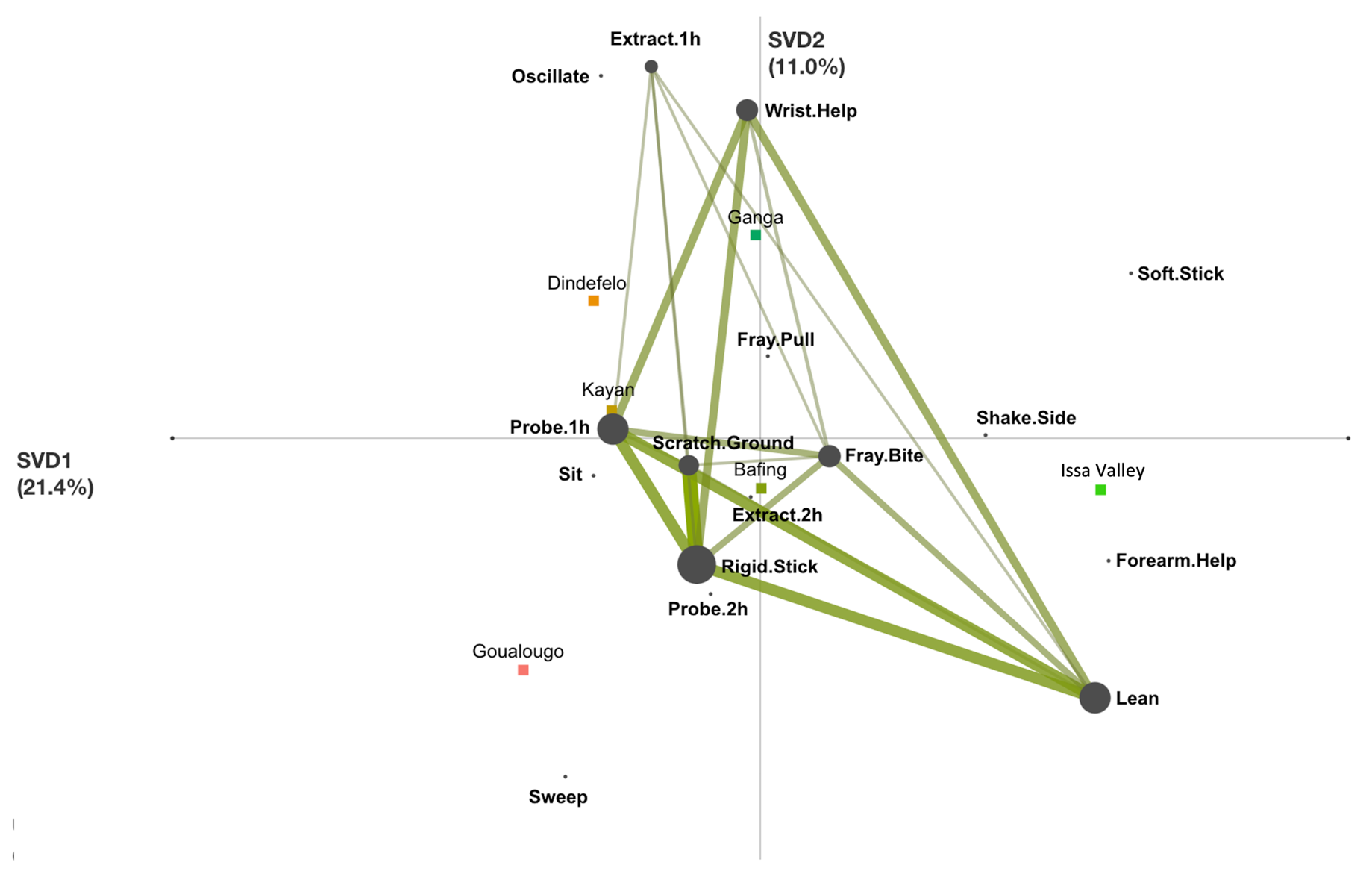 |
| --- |
| Figure S7. Epistemic network of termite fishing behavioral elements in the Bafing chimpanzee community at aerial termite mounds. This figure shows the connections between behavioral elements (circles) for Bafing chimpanzees. Stronger connections are represented by thicker lines. Variance explained by the x-dimension = 21.4%, Variance explained by the y-dimension=11.0%. Squares represent unit means for each community. |


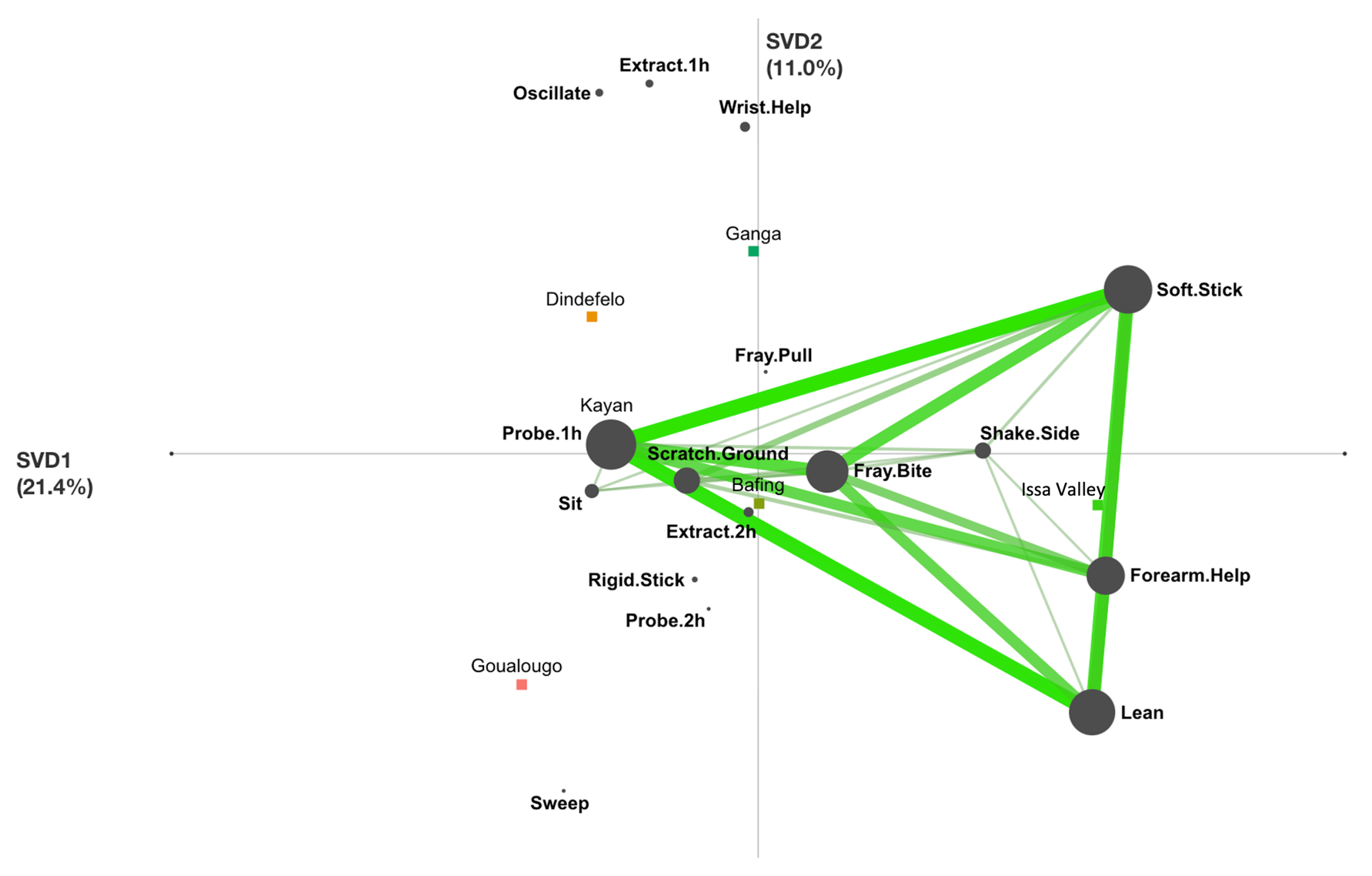
Figure S8. Epistemic network of termite fishing behavioral elements in the Issa Valley chimpanzee community at aerial termite mounds. This figure shows the connections between behavioral elements (circles) for Issa Valley chimpanzees. Stronger connections are represented by thicker lines. Variance explained by the x-dimension = 21.4%, Variance explained by the y-dimension=11.0%. Squares represent unit means for each community.

| 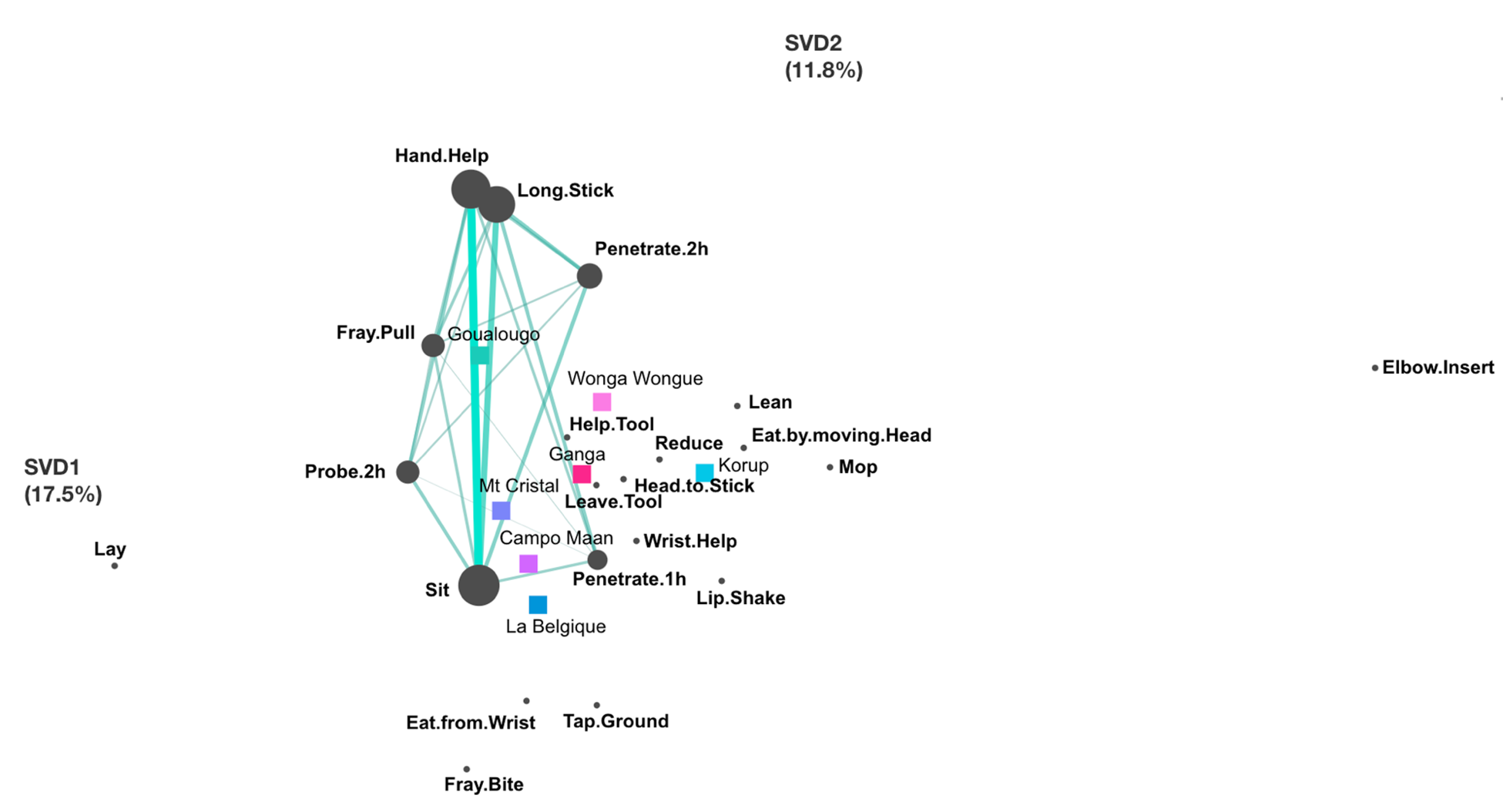 |
| --- |
| Figure S9. Epistemic network of termite fishing behavioral elements in the Goualougo chimpanzee community at underground termite mounds. This figure shows the connections between behavioral elements (circles) for Goualougo chimpanzees. Stronger connections are represented by thicker lines. Variance explained by the x-dimension = 17.5%, Variance explained by the y-dimension=11.8%. Squares represent unit means for each community. |

| 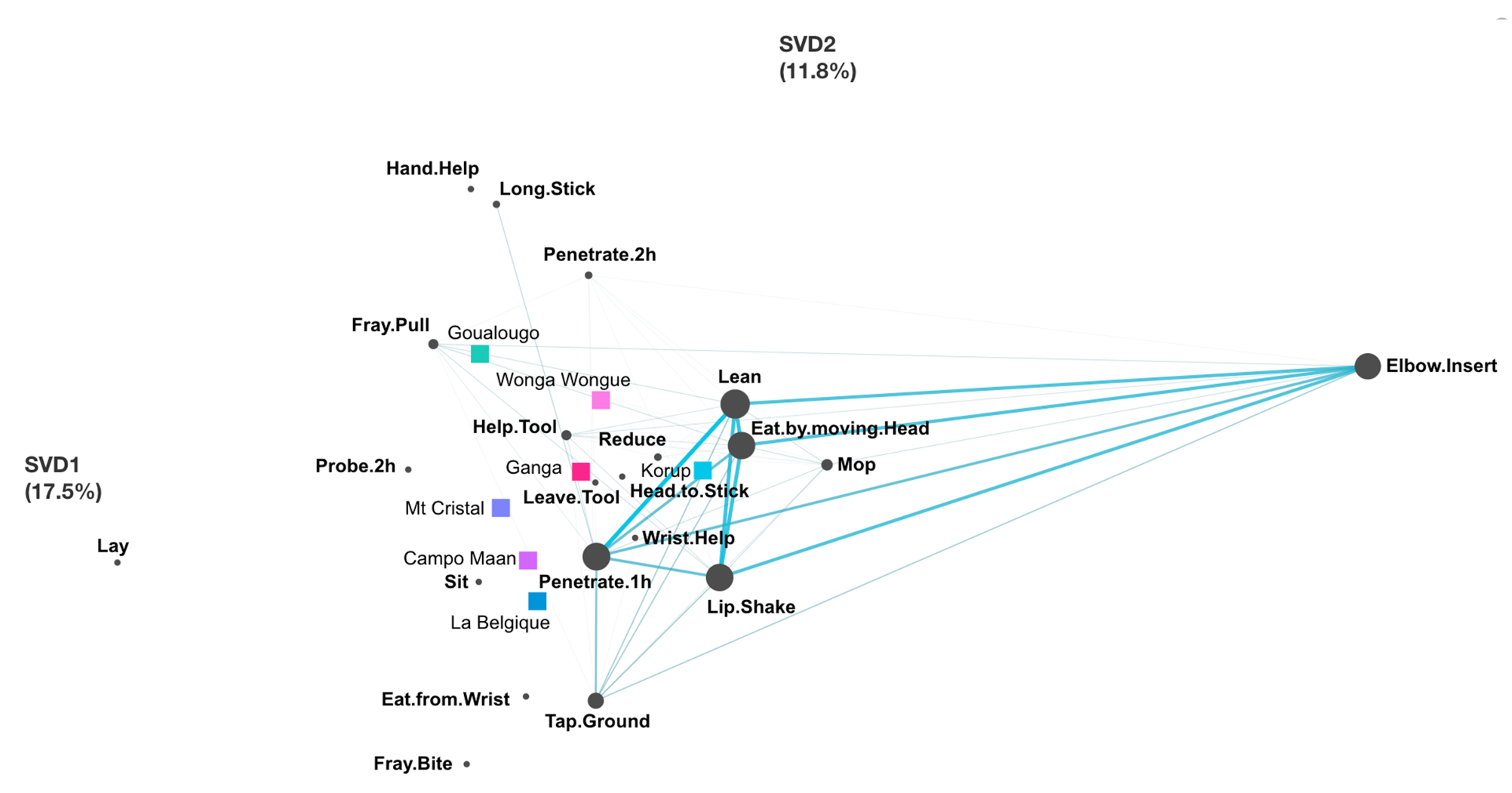 |
| --- |
| Figure S10. Epistemic network of termite fishing behavioral elements in the Korup chimpanzee community at underground termite mounds. This figure shows the connections between behavioral elements (circles) for Korup chimpanzees. Stronger connections are represented by thicker lines. Variance explained by the x-dimension = 17.5%, Variance explained by the y-dimension=11.8%. Squares represent unit means for each community. |

| 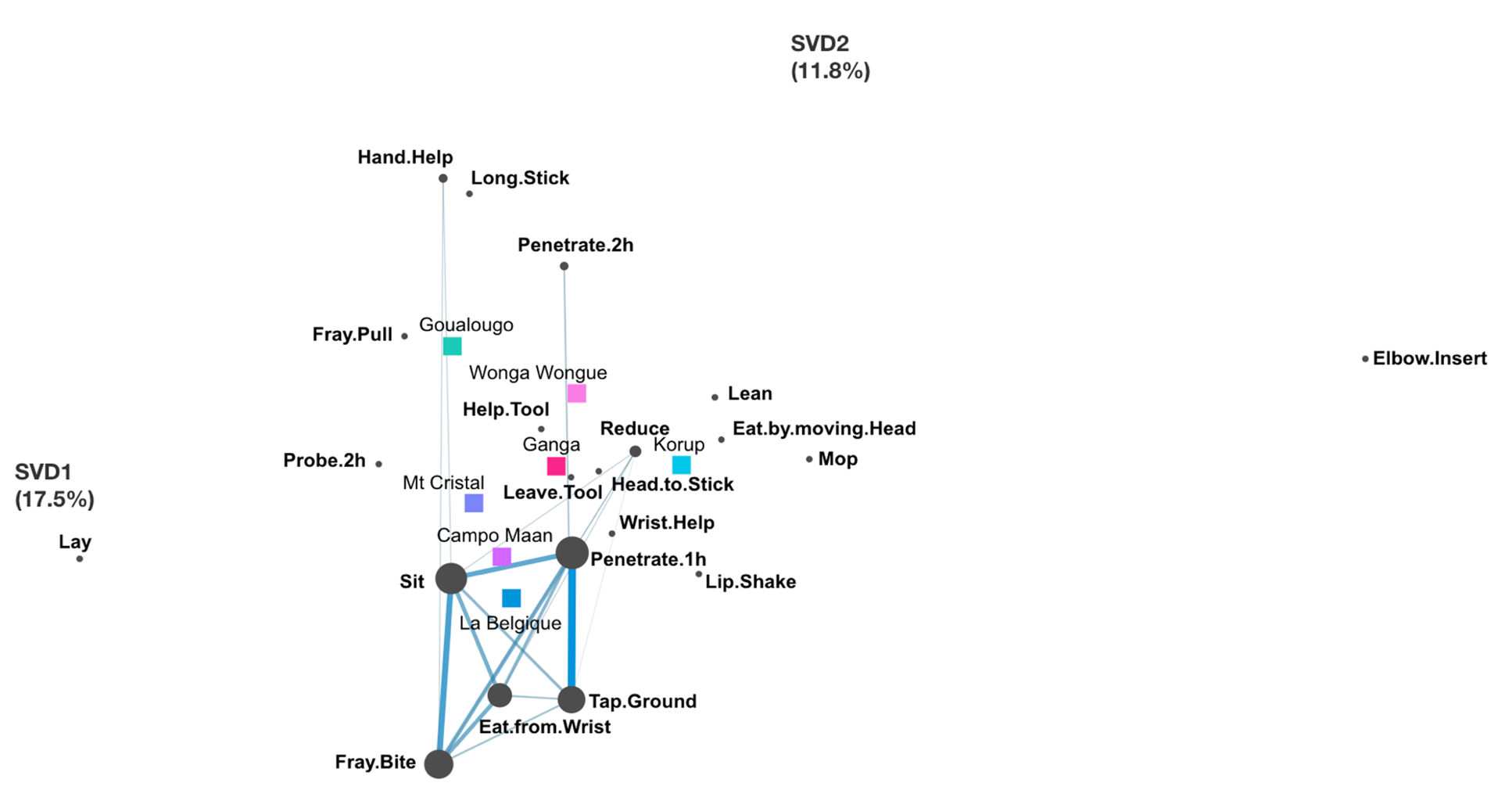 |
| --- |
| Figure S11. Epistemic network of termite fishing behavioral elements in the La Belgique chimpanzee community at underground termite mounds. This figure shows the connections between behavioral elements (circles) for La Belgique chimpanzees. Stronger connections are represented by thicker lines. Variance explained by the x-dimension = 17.5%, Variance explained by the y-dimension=11.8%. Squares represent unit means for each community. |

| 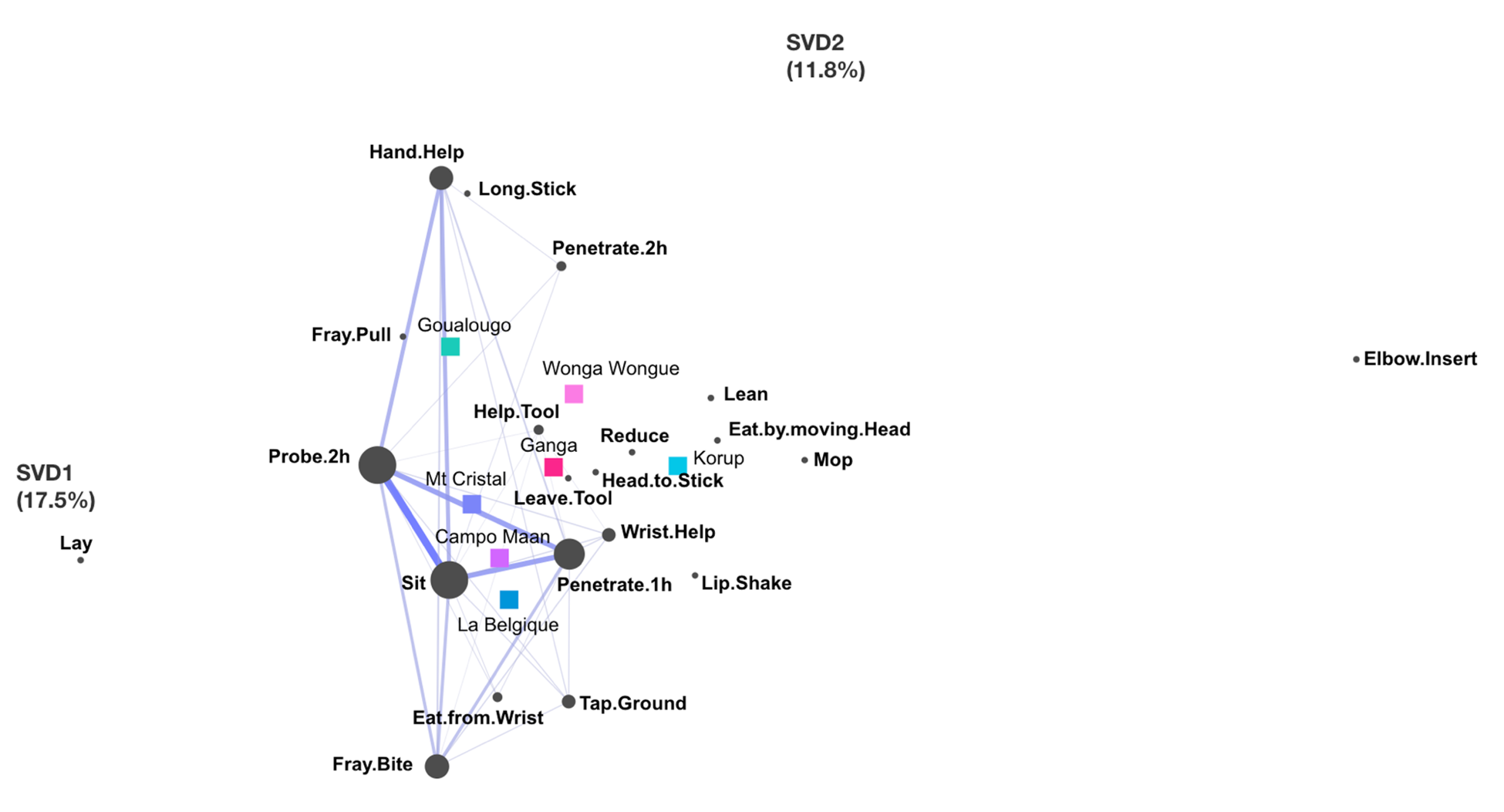 |
| --- |
| Figure S12. Epistemic network of termite fishing behavioral elements in the Mt. Cristal chimpanzee community at underground termite mounds. This figure shows the connections between behavioral elements (circles) for Mt. Cristal chimpanzees. Stronger connections are represented by thicker lines. Variance explained by the x-dimension = 17.5%, Variance explained by the y-dimension=11.8%. Squares represent unit means for each community. |

| 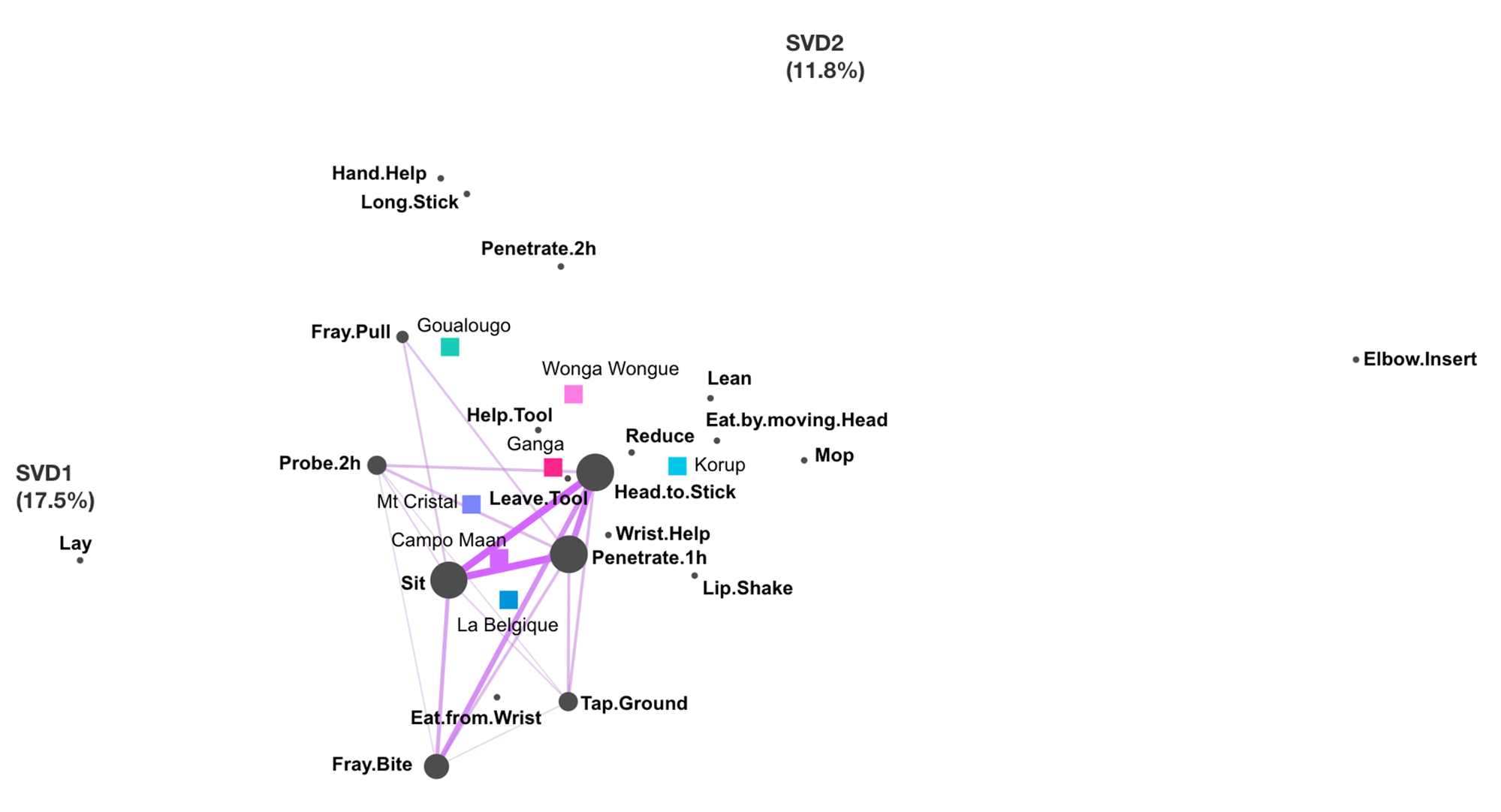 |
| --- |
| Figure S13. Epistemic network of termite fishing behavioral elements in the Campo Ma’an chimpanzee community at underground termite mounds. This figure shows the connections between behavioral elements (circles) for Campo Ma’an chimpanzees. Stronger connections are represented by thicker lines. Variance explained by the x-dimension = 17.5%, Variance explained by the y-dimension=11.8%. Squares represent unit means for each community. |

| 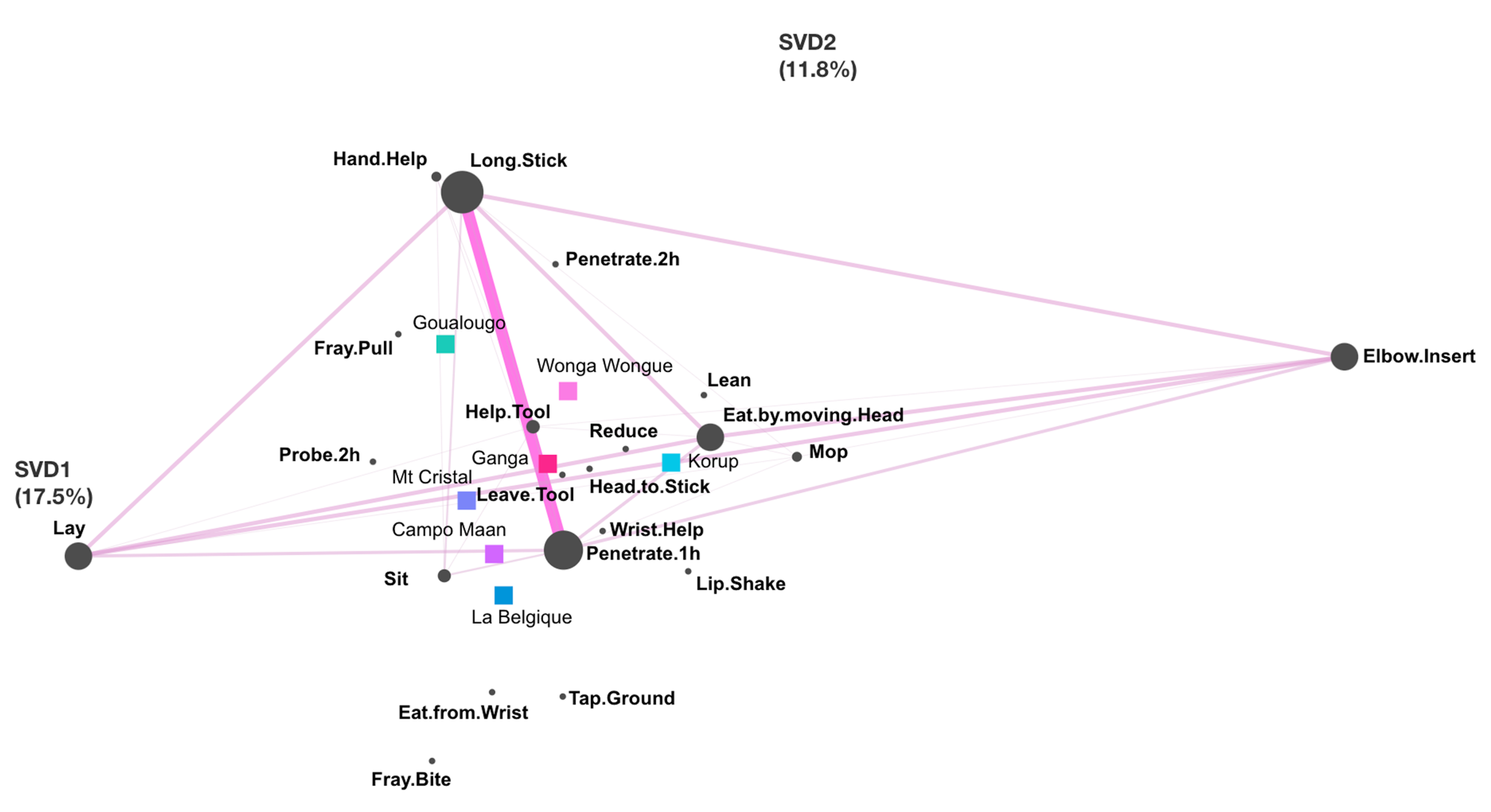 |
| --- |
| Figure S14. Epistemic network of termite fishing behavioral elements in the Wonga-Wongué chimpanzee community at underground termite mounds. This figure shows the connections between behavioral elements (circles) for Wonga-Wongué chimpanzees. Stronger connections are represented by thicker lines. Variance explained by the x-dimension = 17.5%, Variance explained by the y-dimension=11.8%. Squares represent unit means for each community. |
